# Behavioral, neuroanatomical, and molecular correlates of resilience and susceptibility to maternal immune activation

**DOI:** 10.1038/s41380-020-00952-8

**Published:** 2020-11-23

**Authors:** Flavia S. Mueller, Joseph Scarborough, Sina M. Schalbetter, Juliet Richetto, Eugene Kim, Amalie Couch, Yohan Yee, Jason P. Lerch, Anthony C. Vernon, Ulrike Weber-Stadlbauer, Urs Meyer

**Affiliations:** 1 Institute of Pharmacology and Toxicology, University of Zurich-Vetsuisse, Zurich, Switzerland; 2 Neuroscience Center Zurich, University of Zurich and ETH Zurich, Zurich, Switzerland; 3 Department of Neuroimaging, Institute of Psychiatry, Psychology and Neuroscience, King’s College London, London, UK; 4 Department of Basic and Clinical Neuroscience, Institute of Psychiatry, Psychology and Neuroscience, King’s College London, London, UK; 5 Mouse Imaging Centre, Hospital for Sick Children, Toronto, ON Canada; 6 Wellcome Centre for Integrative Neuroimaging, University of Oxford, Oxford, UK; 7 Department of Medical Biophysics, University of Toronto, Toronto, Canada; 8 MRC Centre for Neurodevelopmental Disorders, King’s College London, London, UK

**Keywords:** Neuroscience, Schizophrenia

## Abstract

Infectious or noninfectious maternal immune activation (MIA) is an environmental risk factor for psychiatric and neurological disorders with neurodevelopmental etiologies. Whilst there is increasing evidence for significant health consequences, the effects of MIA on the offspring appear to be variable. Here, we aimed to identify and characterize subgroups of isogenic mouse offspring exposed to identical MIA, which was induced in C57BL6/N mice by administration of the viral mimetic, poly(I:C), on gestation day 12. Cluster analysis of behavioral data obtained from a first cohort containing >150 MIA and control offspring revealed that MIA offspring could be stratified into distinct subgroups that were characterized by the presence or absence of multiple behavioral dysfunctions. The two subgroups also differed in terms of their transcriptional profiles in cortical and subcortical brain regions and brain networks of structural covariance, as measured by ex vivo structural magnetic resonance imaging (MRI). In a second, independent cohort containing 50 MIA and control offspring, we identified a subgroup of MIA offspring that displayed elevated peripheral production of innate inflammatory cytokines, including IL-1β, IL-6, and TNF-α, in adulthood. This subgroup also showed significant impairments in social approach behavior and sensorimotor gating, whereas MIA offspring with a low inflammatory cytokine status did not. Taken together, our results highlight the existence of subgroups of MIA-exposed offspring that show dissociable behavioral, transcriptional, brain network, and immunological profiles even under conditions of genetic homogeneity. These data have relevance for advancing our understanding of the variable neurodevelopmental effects induced by MIA and for biomarker-guided approaches in preclinical psychiatric research.

## Introduction

Infectious or noninfectious maternal immune activation (MIA) is an environmental risk factor for psychiatric and neurological disorders with neurodevelopmental etiologies [1]. Various pathophysiological processes contribute to the association between MIA and neurodevelopmental disorders, including inflammation and oxidative stress occurring in maternal and fetal compartments, activation of maternal stress response systems, temporary micro- and/or macronutrient deficiencies, disruption of placental functions, and epigenetic remodeling of the offspring’s molecular machinery [2, 3]. When occurring during sensitive periods of fetal brain development, these pathophysiological processes have the potential to change the offspring’s neurodevelopmental trajectories and increase their risk to develop psychiatric and neurological disorders in later life [1–3].

Despite the increasing evidence for significant health consequences, it is important to note that the effects of MIA on offspring are variable [4]. While some offspring of MIA-exposed mothers develop central nervous system (CNS) disorders, a substantial portion does not [1, 5]. Hence, there may be a considerable degree of resilience to MIA, which determines the extent to which offspring are protected from developing lasting abnormalities in brain functions and behavior. Several antenatal factors are emerging as elements that can promote resilience to MIA [4], including low maternal baseline immunoreactivity [6]. The latter has recently been identified in an isogenic mouse model of MIA and extends findings from epidemiological and experimental investigation suggesting that the intensity of MIA is a critical factor determining the neurodevelopmental consequences in the offspring [7–11].

The genetic background is also likely to be a major determinant of the severity and/or specificity of the neurodevelopmental sequela of MIA [12, 13]. Indeed, genetic factors can promote resilience [14] or susceptibility to MIA [15, 16], either through their influence on host defense and immunity or by concurrently affecting neurodevelopmental programs independently of MIA [4]. Whereas the investigation of gene–environment interactions in the context of MIA is a highly active field of research [13, 17–19], it remains largely unknown how MIA can lead to phenotypic variability under conditions of genetic homogeneity [4].

Here, we identify and characterize subgroups of isogenic mouse offspring with distinct clusters of behavioral symptoms emerging after exposure to identical MIA, which was induced by maternal administration of poly(I:C) during pregnancy. Poly(I:C) is a synthetic analog of double-stranded RNA that induces a cytokine-associated viral-like acute phase response [20] and is widely used in immune-mediated neurodevelopmental disruption models as applied to mice [21, 22] and other species [23]. Using the poly(I:C)-based MIA model in mice, we demonstrate that offspring with manifest behavioral anomalies at adult age differ from asymptomatic offspring in terms of their transcriptional profiles in cortical and subcortical brain regions and brain networks of structural covariance, while displaying only subtle differences in regional brain volumes, as measured by ex vivo structural magnetic resonance imaging (MRI). Furthermore, we show that adult offspring of MIA-exposed mothers can be stratified into subgroups with distinct plasma cytokine profiles, which in turn predict the presence or absence of MIA-induced behavioral anomalies.

## Methods

### Animals, breeding, and maternal manipulations

C57BL6/N mice (Charles River Laboratories, Sulzfeld, Germany) were used throughout the whole study. Three independent cohorts of timed-pregnant mice (Supplementary Table S1 in Supplement 1) were generated via on-site breeding. Whereas cohort 1 was used for large-scale behavioral phenotyping and subsequent transcriptional profiling and MRI, cohort 2 was used for measurements of peripheral cytokines, followed by behavioral analyses (Supplementary Table S1 in Supplement 1). Cohort 3 was used to correlate maternal changes in thermoregulation with behavioral profiles in the adult offspring (Supplementary Table S1 in Supplement 1).

All three cohorts were generated under identical experimental conditions as described in Supplement 1. On gestational day (GD) 12, pregnant mice were randomly assigned to a single injection of poly(I:C) (potassium salt, P9582, Sigma–Aldrich, Buchs, St. Gallen, Switzerland) or treatment with endotoxin-free 0.9% NaCl (B. Braun, Melsungen, Switzerland) vehicle solution. Methodological details regarding the MIA model, including birth conditions and weaning of offspring, can be obtained in Supplement 1 and Supplement 3. The latter provides a reporting guideline checklist for the MIA model [21].

### Behavioral testing

Behavioral testing commenced when the offspring reached 12 weeks of age and included tests for innate anxiety-like behavior and locomotor activity (open field test), working memory (spontaneous alternation in the Y-maze), social interaction (a modified version of the three-chamber social interaction test), and PPI of the acoustic startle reflex. Whenever subjected to behavioral testing, each offspring was tested repeatedly using the same order of testing (1. open field test; 2. Y-maze working memory test; 3. social interaction test; 4. PPI test), with a resting phase of 3–4 days between individual tests. A detailed description of the methodological procedures and rationale of inclusion are provided for each behavioral test in Supplement 1.

### Molecular and neuroanatomical analyses

Transcriptomic profiles were assessed in the medial prefrontal cortex (mPFC) and amygdala (Amy), two brain regions with broad relevance for neurodevelopmental disorders [24–26], using next-generation RNA sequencing and gene network analysis as described in Supplement 1. Neuroanatomical analyses were performed using whole-brain ex vivo structural MRI as described in Supplement 1.

### Cytokine measurements

Protein levels of interleukin (IL)-1β IL-6, IL-10, tumor necrosis factor (TNF)-α and interferon (IFN)- γ were measured in the plasma of pregnant mice and adult offspring using a customized Meso-Scale Discovery (MSD) V-Plex electrochemiluminescence assay (MSD, Rockville, MA, USA) for mice as described in Supplement 1.

### Statistical analyses

A detailed description of the statistical analyses is provided in Supplement 1.

## Results

### Variability in MIA-induced behavioral changes

**Fig. 1:**
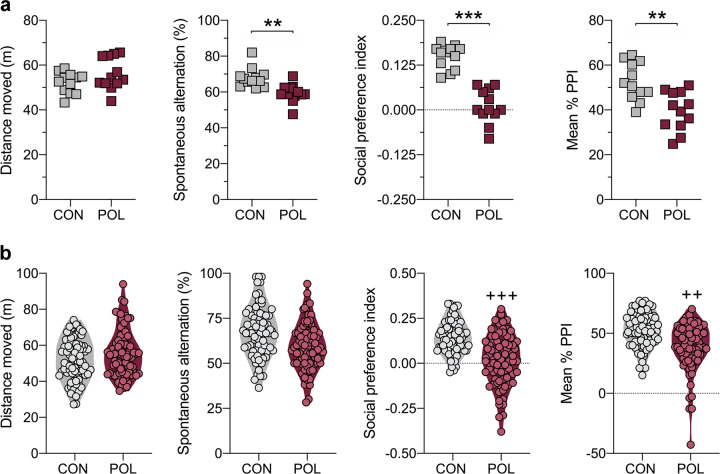
Main behavioral performance of MIA-exposed and control offspring. Pregnant C57BL6/N mice were exposed to poly(I:C) (POL) or control (CON) treatment on gestation day 12. At adult age (12 weeks onwards), the resulting offspring were subjected to a behavioral testing battery assessing basal locomotor activity in the open field test, working memory in a Y-maze spontaneous alternation test, sociability in a social interaction test (social preference index values >0 represent a preference toward an unfamiliar mouse, whereas values <0 represent a preference towards an inanimate dummy object), and prepulse inhibition (PPI) of the acoustic startle reflex. **a** Litter-based analyses of the main behavioral data, in which the number of litters (*N* = 12 per treatment group) was considered as the  experimental unit. The scatter plots show the distance moved (m) in the open field test, spontaneous alternation (%) in the Y-maze working memory test (*t*_(22)_ = 3.38, **p < 0.01), social preference index in the social interaction test (*t*_(22)_ = 5.98, ***p < 0.001), and the mean % PPI in the PPI test of the acoustic startle reflex (*t*_(22)_ = 3.75, **p < 0.01). **b** The violin plots with overlaid data points show the performance of individual CON (*n* = 77, from *N* = 12 litters) and POL (*n* = 81, from *N* = 12 litters) offspring in the four behavioral tests. Compared to the CON group, the POL group shows significantly larger dispersion of data relating to social approach behavior (*F*_(80,76)_ = 2.29, ^+++^*p* < 0.001) and mean %PPI (*F*_(80,76)_ = 1.88, ^++^*p* < 0.01), based on *F*-tests to compare variances.

We generated a cohort of MIA-exposed and control offspring by administering the viral mimetic poly(I:C) (POL) and vehicle solution (CON), respectively, to isogeneic C57BL6/N mice on GD 12. Using validated procedures [11, 27], this cohort was produced by a single on-site breeding and encompassed 12 CON litters with 77 offspring and 12 POL litters with 81 offspring (Supplementary Table S1 in Supplement 1). Once the offspring reached adulthood (12 weeks of age onwards), all offspring were assigned to behavioral phenotyping. When conducting litter-based analyses of the behavioral data, in which the number of litters (*N* = 12 per treatment group) was considered as the experimental unit, we found that the group of POL offspring displayed significant impairments in working memory, social approach behavior, and PPI (Fig. 1a). These effects were not confounded by  possible changes in basal locomotor activity (Fig. 1a, Supplementary Fig. S1b in Supplement 2), general object exploration in the social interaction test (Supplementary Fig. S1c in Supplement 2), or baseline startle reactivity (Supplementary Fig. S1d in Supplement 2). There were also no significant group differences in terms of indices of innate anxiety (Supplementary Fig. S1a in Supplement 2).

Examining individual offspring in the CON (*n* = 77) and POL (*n* = 81) groups revealed a marked dispersion of their behavioral scores. As summarized in Fig. 1b, the dispersion of data relating to social approach behavior and PPI was significantly larger in the POL group compared to the CON group. There was also a large dispersion of data relating to the distance moved in the open field and working memory scores in the Y-maze, with comparable magnitudes between CON and POL offspring (Fig. 1b). Hence, despite the significant group differences revealed by litter-based analyses (Fig.1a), the effects of MIA in this model system appear variable when considering individual offspring from multiple independent litters.

### Stratification into resilient and susceptible subgroups based on behavioral profiles

The variable behavioral phenotypes induced by MIA in the poly(I:C)-based model system prompted us to explore whether there are subgroups of offspring that can be stratified based on their behavioral profiles. To this aim, we performed a two-step cluster analysis that integrated the main behavioral measures (total distance moved in the open field, spontaneous alternation in the Y-maze test of working memory, social preference index obtained in the social interaction test, and PPI of the acoustic startle reflex) from each individual CON and POL offspring. To avoid bias in our data set, we did not predetermine the possible number of clusters [28].

**Fig. 2:**
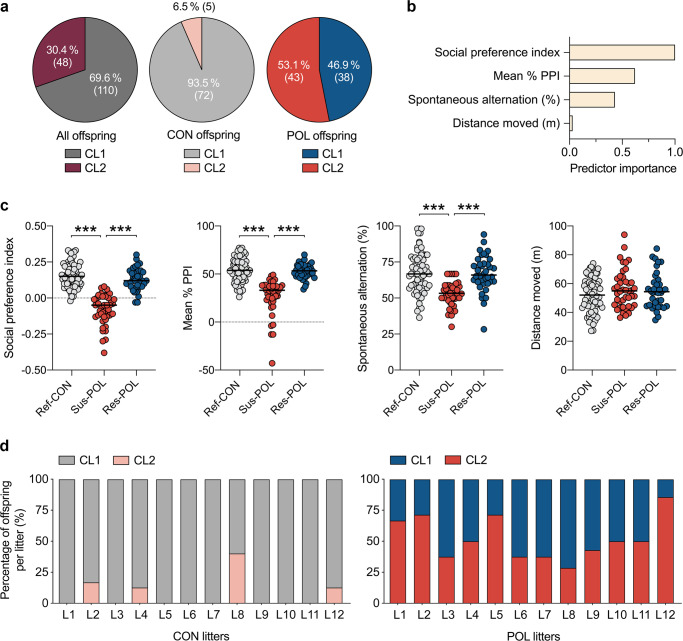
Stratification of MIA-exposed offspring into resilient and susceptible subgroups. A two-step cluster analysis incorporating the main behavioral measures (total distance moved in the open field, spontaneous alternation in the Y-maze test of working memory, social preference index in the social interaction test, and mean % PPI of the acoustic startle reflex) from individual control (CON; *n* = 77, originating from 12 litters) and poly(I:C)-exposed (POL; *n* = 81, originating from 12 litters) offspring was performed to identify subgroups with differing behavioral profiles. **a** Distribution of CON and POL offspring across the two clusters (CL1 and CL2) identified by two-step cluster analysis. The pie charts show the cluster distribution (in percentages, %) for all offspring combined, and for CON and POL offspring separately. The numbers in brackets represent the number of offspring in each cluster. **b** Summary of the relative predictor importance for cluster separation as revealed by two-step cluster analysis. **c** The scatter plots show the main behavioral readouts for subgroups of CON and POL offspring as identified by two-step cluster analysis. Compared to reference CON (Ref-CON, *n* = 72) and resilient POL (Res-POL, *n* = 38) offspring, susceptible POL (Sus-POL, *n* = 43) display a significant reduction in the social preference index (*F*_(2,150)_ = 101.0, *p* < 0.001; Sus-POL vs. Ref-CON or Res-POL: ***p < 0.001), mean % PPI in the PPI test of the acoustic startle reflex (*F*_(2,150)_ = 40.8, *p* < 0.001; Sus-POL vs. Ref-CON or Res-POL: ***p < 0.001) and spontaneous alternation in the Y-maze working memory test (*F*_(2,150)_ = 22.3, *p* < 0.001; Sus-POL vs. Ref-CON or Res-POL: ***p < 0.001), based on ANOVA and Tukey’s post hoc tests. **d** Distribution of CL1 and CL2 offspring across CON and POL litters (*N* = 12 for each treatment, L1–L12). Note that each POL litter concomitantly contained offspring identified as belonging to CL1 (representing Res-POL offspring) and CL2 (representing Sus-POL offspring).

The two-step cluster analysis identified two main clusters (CL1 and CL2) with good cluster separation (silhouette measure of cohesion and separation >0.6). A total of 110 offspring were identified as belonging to CL1, whereas 48 offspring were classified into CL2 (Fig. 2a). The majority of CON offspring (93.5%, 72 out of 77) were assigned to CL1 (Fig. 2a). By contrast, only 46.9% (38 out of 81) of the POL offspring were identified as belonging to CL1, whereas the remaining (53.1%; 43 out of 81) were classified into CL2 (Fig. 2a). Hence, the CL1/CL2 ratio differed significantly (*χ*^2^ = 40.52, *z* = 6.37, *p* < 0.001) between CON and POL offspring. As shown in Fig. 2b, the social preference index obtained from the social interaction test had the highest predictor importance for cluster separation, followed by PPI of the acoustic startle reflex and spontaneous alternation in the Y-maze test of working memory. Total distance moved in the open field had only minimal predictor importance for cluster separation (Fig. 2b).

In general, offspring classified into CL1 showed a higher social preference index, higher mean % PPI scores, and higher levels of spontaneous alternation in the Y-maze test of working memory than offspring identified as belonging to CL2. Therefore, we termed CON-CL1 and POL-CL1 offspring as “reference” CON (Ref-CON) and “resilient” POL (Res-POL) offspring, respectively, whereas we referred POL-CL2 offspring to as “susceptible” to MIA (Sus-POL) offspring. The subsequent comparison of Ref-CON, Sus-POL, and Res-POL subgroups confirmed that only Sus-POL, but not Res-POL offspring, displayed significant deficits in spontaneous alternation in the Y-maze, social preference index and mean % PPI compared to Ref-CON offspring (Fig. 2c), while all behavioral measures were largely identical between the Res-POL and Ref-CON subgroups (Fig. 2c).

Contrasting between- and within-litter effects further demonstrated that Res-POL and Sus-POL were distributed consistently across all POL litters (Fig. 2d). Hence, each POL litter concomitantly contained Res-POL and Sus-POL, indicating that the stratification of the two subgroups is mainly driven by within-litter rather than between-litter variability. To further probe the contribution of between-litter variability arising from variations in the maternal response to MIA, we correlated maternal changes in thermoregulation after CON or POL exposure with the behavioral profiles in the adult offspring (Supplementary Fig. S2 in Supplement 2). These investigations showed that MIA-induced maternal hypothermia, which correlated with the maternal IL-6 response to poly(I:C), did not predict the behavioral outcomes in the adult offspring (Supplementary Fig. S2 in Supplement 2). These data corroborate the hypothesis that between-litter variability arising from variations in the maternal response to MIA plays a minor role in the dissociation of offspring into susceptible and resilient subgroups, at least under the present experimental conditions.

When conducting the two-step cluster analysis separately for male (*n* = 39 for CON, *n* = 46 for POL) and female (*n* = 38 for CON, *n* = 35 for POL) offspring, we yielded results that were consistent with the stratification of the entire cohort of offspring containing both sexes. As summarized in Supplementary Fig. S3 (Supplement 2), the cluster analyses revealed two main clusters (CL1 and CL2) in both male and female offspring. The CL1/CL2 (resilient/susceptible) ratio appeared to differ between male (45.6% in CL1 vs. 54.4% in CL2; Supplementary Fig. S3a in Supplement 2) and female (25.7% in CL1 vs. 74.3% in CL2; Supplementary Fig. S3c in Supplement 2) POL offspring. Hence, when the cluster analysis was conducted separately for either sex, a higher percentage of females (74.3%) was assigned to CL2 (i.e., the “susceptible cluster”) as compared to males (54.4%). At the same time, however, more female (10.5%; 4 out of 38) than male (2.6%; 1 out of 39) CON offspring were allocated to CL2. Together these data show that the cluster analysis of behavioral data generally assigned a larger portion of females than male offspring to CL2, and accordingly, the *χ*^2^-distribution of CL2/CON and CL2/POL offspring in either sex failed to reveal statistical significance (*χ*^2^ = 1.54, *z* = 1.24, *p* = 0.21). Likewise, comparing the relative amount of CL1/POL and CL2/POL offspring in either sex did not yield statistical significance (*χ*^2^ = 3.38, *z* = 1.84, *p* = 0.07). For either sex, CL2/POL ( = Sus-POL) offspring displayed a lower social preference index, lower spontaneous alternation in the Y-maze test of working memory and lower PPI of the acoustic startle reflex compared to CL1/POL ( = Res-POL) offspring of the corresponding sex. Consistent with the cluster analysis integrating both sexes simultaneously (Fig. 2b), the social preference index had the highest predictor importance for cluster separation when the cluster analysis was performed separately for male and female offspring (Supplementary Fig. S3 in Supplement 2).

### Transcriptomic correlates of resilience and susceptibility to MIA

The presence of subgroups of MIA-exposed offspring with or without overt behavioral anomalies provides the unique opportunity to identify molecular correlates of resilience and susceptibility to MIA [28]. Therefore, we performed next-generation RNA sequencing to compare genome-wide transcriptional changes in the mPFC and Amy of a subset of Ref-CON (*n* = 3), Sus-POL (*n* = 3), and Res-POL (*n* = 3) offspring that were behaviorally characterized and stratified in the initial large-scale cluster analysis (Fig. 2; Supplementary Table 1 in Supplement 1). Since a similar stratification of MIA-exposed offspring into susceptible and resilient subgroups was obtained for males and females (Supplementary Fig. S3 in Supplement 2), RNA sequencing was conducted only for one sex (males).

**Fig. 3:**
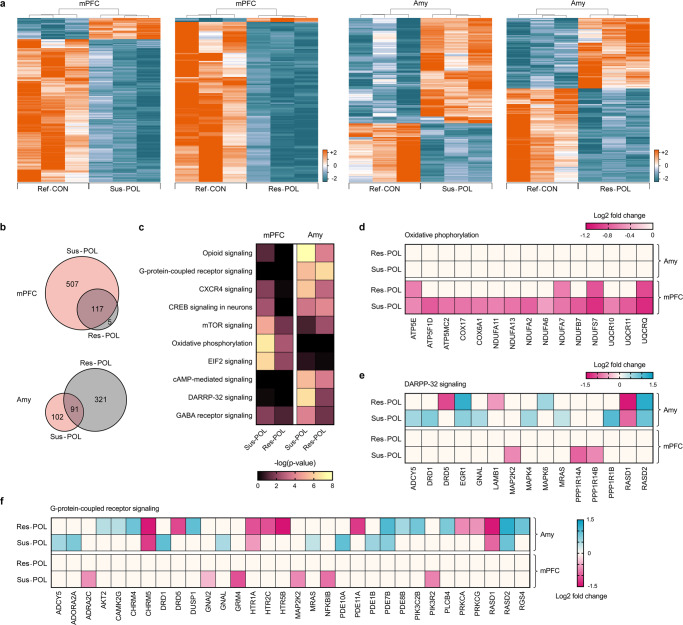
Transcriptional profiles of resilient and susceptible MIA-exposed offspring. Next-generation RNA sequencing was conducted in the medial prefrontal cortex (mPFC) and amygdala (Amy) of resilient (Res-POL) and susceptible (Sus-POL) MIA-exposed offspring, which were stratified based on their behavioral performance (see Fig. 3). Behaviorally characterized reference control (Ref-CON) offspring were used as a comparison. **a** Heat maps illustrating the clustering of differentially expressed genes (FDR: *q* < 0.05) in the mPFC or Amy of Sus-POL or Res-POL offspring relative to Ref-CON offspring. The color-coded key denotes upregulation (orange) and downregulation (blue) in terms of log2 fold changes. **b** Venn diagram denoting the number of genes that are uniquely and commonly affected in the mPFC or Amy of Sus-POL and Res-POL offspring. **c** Heat map of the top ten signaling pathways affected in the mPFC or Amy of Sus-POL and Res-POL offspring, as identified by Ingenuity Pathway Analysis (IPA). The color-coded key denotes the significance levels in terms of the −log(*p* value). **d** Heat map of individual genes annotated with the term “oxidative phosphorylation”. The color-coded key denotes the level of downregulation (magenta tones) in the mPFC or Amy of Sus-POL and Res-POL offspring. **e** Heat map of individual genes annotated with the term “DARPP-32 signaling”. The color-coded key denotes the level of upregulation (blue tones) or downregulation (magenta tones) in the mPFC or Amy of Sus-POL and Res-POL offspring. **f** Heat map of individual genes annotated with the term “G-protein-coupled receptor signaling”. The color-coded key denotes the level of upregulation (blue tones) or downregulation (magenta tones) in the mPFC or Amy of Sus-POL and Res-POL offspring.

Using a false-discovery rate (FDR) threshold of *q* < 0.05, we identified differentially expressed genes (DEGs) in Sus-POL relative to Ref-CON offspring, as well as in Res-POL relative to Ref-CON offspring. The DEGs clustered according to subgroups in both mPFC and Amy (Fig. 3a). We identified 624 DEGs (81 upregulated, 543 downregulated) in the mPFC of Sus-POL offspring relative to Ref-CON offspring, whereas Res-POL offspring only displayed 122 DEGs (3 upregulated, 119 downregulated) in this region (Fig. 3b). 117 DEGs in the mPFC were common to the two subgroups, whereas 507 and 5 DEGs were specifically found in Sus-POL and Res-POL offspring, respectively (Fig. 3b). Hence, Sus-POL offspring showed quantitative more DEGs in the mPFC than Res-POL offspring. On the contrary, Res-POL offspring displayed a larger number of DEGs in Amy as compared to Sus-POL offspring. While the Res-POL subgroup showed 412 DEGs (177 upregulated, 235 downregulated) in this region, only 193 DEGs (124 upregulated, 69 downregulated) were identified in the Amy of Sus-POL offspring (Fig. 3b). Totally, 91 DEGs in the Amy were common to the two subgroups, whereas 102 and 321 DEGs were exclusively present in Sus-POL and Res-POL offspring, respectively (Fig. 3b).

We used Ingenuity Pathway Analysis (IPA) to identify canonical signaling pathways affected in the two subgroups of MIA-exposed offspring relative to Ref-CON offspring. The top ten of these pathways is shown in Fig. 3c, whereas the corresponding DEGs annotated with each pathway are provided in Supplementary Table S2 (Supplement 2). In addition to altering neuronal signaling pathways such as dopamine- and cAMP-regulated phosphoprotein 32 kDa (DARPP-32) signaling, γ-aminobutyric acid (GABA) receptor signaling, and opioid signaling, MIA affected gene sets annotating with mitochondrial oxidative phosphorylation and translation initiation by eukaryotic initiation factor 2 (EIF2) signaling (Fig. 3c, Supplementary Table S2 in Supplement 2). Notably, the extent to which MIA affected these pathways was dependent on the brain region and markedly differed between Sus-POL and Res-POL offspring. For example, a larger number of genes annotating with oxidative phosphorylation was downregulated in the mPFC of Sus-POL offspring compared to Res-POL offspring, whereas the Amy of both subgroups did not show similar transcriptional changes in the oxidative phosphorylation pathway (Fig. 3d). By contrast, genes pertaining to DARPP-32 signaling were more strongly affected by MIA in the Amy than in the mPFC, with individual DEGs largely differing between Sus-POL and Res-POL offspring (Fig. 3e). Similar brain region- and gene-specific effects were also notable for genes annotating with G-protein-coupled receptor signaling (Fig. 3f).

A direct contrast between the FDR-corrected (*q* < 0.05) transcriptional profiles of Sus-POL and Res-POL offspring further identified reduced mRNA levels of several genes annotated with the term “neuronal activity-regulated gene transcription”, including c-Fos, Arc, and Npas4, in the mPFC of Sus-POL offspring relative to Res-POL offspring (Supplementary Table S3 in Supplement 2). Moreover, the Sus-POL subgroup displayed abnormal amygdalar transcription of genes annotated with the disease and function term “schizophrenia”. Among others, these changes included reduced expression of parvalbumin (Pvalb), erb-b2 receptor tyrosine kinase 3 (Erbb3), synaptotagmin 2 (Syt2), vesicle-associated membrane protein 1 (Vamp1), and disheveled-associated activator of morphogenesis 2 (Daam2) (Supplementary Fig. S4a and Supplementary Table S4 in Supplement 2). We also found that the mRNA levels of several genes annotated with the terms “demyelination”, “abnormal morphology of myelin sheath”, “hypomyelination of axons”, and “abnormal morphology of oligodendrocytes” were reduced in the Amy of Sus-POL offspring relative to Res-POL offspring (Supplementary Fig. S4b and Supplementary Table S4 in Supplement 2). Taken together, these data demonstrate that susceptible and resilient subgroups of MIA-exposed offspring differ in terms of their gene expression profiles in cortical and subcortical brain areas.

### Neuroanatomical correlates of resilience and susceptibility to MIA

Human MRI studies provide evidence for associations between regional brain volumes and connectivity in the offspring and the degree of maternal inflammation during pregnancy, which also mediates behavioral outcomes [29–31]. Using MRI,  we and others have also demonstrated that poly(I:C)-induced MIA results in subtle neuroanatomical changes in the MIA offspring [32–35]. The presence of subgroups of MIA-exposed offspring with or without overt behavioral anomalies, therefore, provides an opportunity to investigate whether this is associated with regional neuroanatomical differences, using a clinically comparable technology (MRI). To test this, we acquired ex vivo structural MRI on fixed brain samples from a subset of Ref-CON (*n* = 16 [8m, 8f]), Sus-POL (*n* = 14 [8m, 6f]), and Res-POL (*n* = 14 [8m, 6f]) offspring that were behaviorally characterized and stratified in the initial large-scale cluster analysis (Fig. 2; Supplementary Table 1 in Supplement 1).

**Fig. 4:**
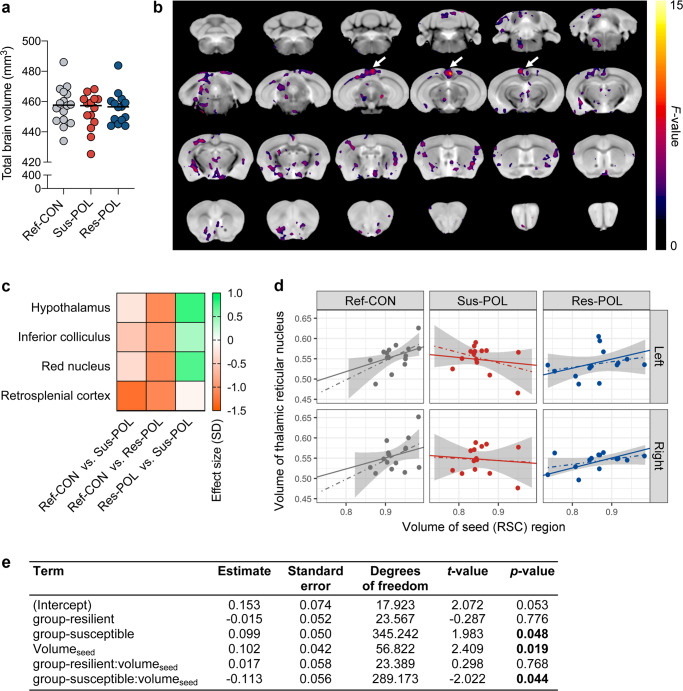
Neuroanatomical and structural covariance characteristics of resilient and susceptible MIA-exposed offspring. Structural magnetic resonance imaging (MRI) was performed ex vivo in resilient (Res-POL) and susceptible (Sus-POL) MIA-exposed offspring, which were stratified based on their behavioral performance (see Fig. 3). Behaviorally characterized reference control (Ref-CON) offspring were used as a comparison. **a** Total brain volume (mm^3^) across groups. **b** MRI fly-through of absolute volume differences across groups. No changes survived stringent correction for multiple comparisons (family wise error rate set at *p* < 0.05). The data shown represent voxel clusters with significant (*p* < 0.05) group differences revealed in exploratory analyses that were uncorrected for multiple comparisons. The clusters of voxels with the largest effect in the retrosplenial cortex (RSC) are highlighted by arrows. **c** Heat map showing effect sizes (in units of SD) for regional volume differences in absolute volume (mm^3^) across each group. Effect sizes are shown for brain areas with significant group differences revealed by uncorrected analyses. **d** Structural covariance between the seed (RSC) and thalamic reticular nucleus (in both hemispheres). For each group, the linear mixed-effects model fit (solid line) is shown, along with a linear model separately fitted for each subgroup/hemisphere (dotted line and shaded area). A similar pattern of positive covariation in Ref-CON offspring, and loss of covariation in Sus-POL offspring, is also seen in other brain structures (see Supplementary Fig. S7). **e** Fixed effects for the linear mixed-effects model predicting the volumes of all target structures in the structural covariance analysis. *n*(Ref-CON) = 16 (8m, 8f), *n*(Res-POL) = 14 (8m, 6f), and *n*(Sus-POL) = 14 (8m, 6f).

Total brain volume, as measured by MRI, varied as a function of sex, with female mice generally showing larger brain volumes than males (Supplementary Fig. S5 in Supplement 2). There was, however, no statistically significant main effect of group or interaction between sex and group. Regardless of sex, therefore, Sus-POL offspring do not differ from Res-POL or Ref-CON offspring in terms of total brain volume (Fig. 4a). Based on these data, we used absolute volumes in mm^3^ for all subsequent comparisons, presented with both sexes combined.

Regionally, there were no statistically significant group differences in absolute volumes (mm^3^) across 71-brain atlas regions of interests (ROIs) after correction for multiple comparisons at 5% FDR (*q* < 0.05). In uncorrected exploratory analyses, 6% (4/71) of brain ROIs differed in volume between groups (Supplementary Table S5 in Supplement 2). Of these, the absolute volumes of the retrosplenial cortex (RSC) had the largest effect sizes, with lower volumes in Sus-POL and Res-POL offspring relative to the Ref-CON group (Fig. 4c, Supplementary Table S5 in Supplement 2). In contrast, the absolute volumes of the red nucleus, hypothalamus, and inferior colliculus were reduced to a greater extent in Res-POL offspring as compared to Ref-CON and Sus-POL offspring (Fig. 4c, Supplementary Table S5 in Supplement 2).

Using a complementary voxel-wise analysis, there were also no statistically significant group-level differences in absolute volumes after stringent correction for multiple comparisons with a family-wise error set at *p* < 0.05, confirming the lack of statistically significant volume differences in the FDR-corrected ROI-based analyses. At an exploratory threshold (*p* < 0.05 uncorrected for multiple comparisons), there were diffuse clusters of voxels differing in absolute volume across the groups, with the largest such cluster present in the RSC, consistent with ROI-based analysis (Fig. 4b). Additional voxel clusters with uncorrected group differences were, however, present in the hypothalamus, olfactory nuclei, diagonal band, ventral midbrain, sensory and parietal cortices, the cerebellum, and amygdalar nuclei, extending our ROI analyses (Fig. 4b).

We next explored whether subgroups of MIA-exposed and control offspring differ in structural covariance, that is, the correlated variation in volumes between pairs of brain regions. This analysis was motivated on the basis that structural covariance is associated with both structural [36] and functional brain connectivity [37], with variations in the latter known to be associated with risk and resilience for serious mental illnesses linked to MIA [30, 38]. Structural covariance metrics are also linked to patterns of coordinated gene expression [39] and synchronized neurodevelopment [40–42]. Since the RSC appeared to be the most strongly implicated brain region from our initial brain-wide volumetric analyses (Fig. 4b, c), we selected this as our seed region and examined whether structures that are normally connected to the RSC might also show correlated changes. To this end, we used publicly available viral tracing data from the Allen Institute (Supplementary Fig. S6 and Supplementary Table S6 in Supplement 1) to determine which regions of the brain are normally connected to the cluster of voxels in the RSC that showed volume differences (“seed”) and to examine structural covariance between the seed region and these connected (“target”) regions.

For the target regions (structures connected to the RSC), both volume and structural covariance differences were identified in Sus-POL offspring compared with Res-POL and Ref-CON offspring. For all target structures modeled together, the volumes of these structures are greater in Sus-POL compared to Ref-CON offspring when there is no influence of the seed region, whereas this effect is not present in Res-POL offspring (Fig. 4e). With regards to structural covariance, Ref-CON and Res-POL offspring displayed positive structural covariance between seed and target regions (Fig. 4e). Intriguingly, Sus-POL offspring also showed statistically significant differences in anatomical covariance using the RSC as the seed region, but in the opposite directions (Fig. 4e). Hence, there was reduced covariance in Sus-POL offspring for the same regions that are positively correlated in Ref-CON and Res-POL offspring (Fig. 4e). A graphical representation of the structural covariance differences is provided for the thalamic reticular nucleus in Fig. 4d, and for all structures in Supplementary Fig. S7 in Supplement 2.

### Stratification of offspring based on cytokine profiles in plasma

Whereas our initial large-scale cluster analysis identified subgroups of MIA-exposed offspring based on behavioral readouts (Fig. 2), we further investigated whether a similar stratification could be obtained through analyses of easily accessible peripheral biomarkers. To this end, we focused on a panel of plasma cytokines (IL-1β, IL-6, IL-10, TNF-α, and IFN-γ), which have been used previously to assess signs of peripheral inflammation in major psychiatric disorders such as schizophrenia [43–46] and animal models of MIA [47–51]. Cytokine proteins were quantified in the plasma of an independent cohort (Supplementary Table 1 in Supplement 1) of adult CON (*n* = 17 [8m, 9f], originating from *N* = 4 litters) and POL (*n* = 33 [16m, 17f], originating from *N* = 6 litters) offspring, after which two-step cluster analysis was used to identify possible subgroups [28].

**Fig. 5:**
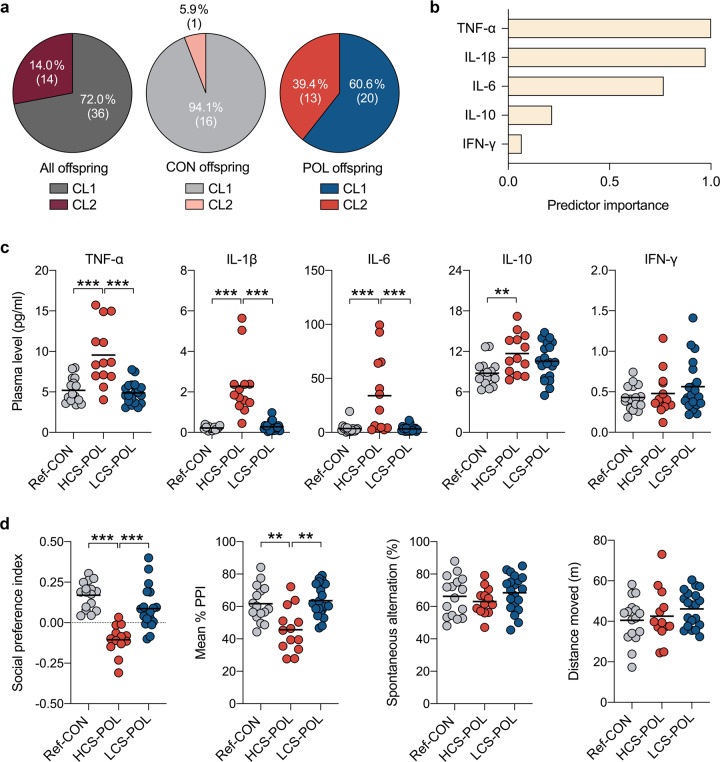
Stratification of MIA-exposed offspring based on plasma cytokine status. A two-step cluster analysis incorporating plasma IL-1β, IL-6, IL-10, TNF-α and IFN-γ protein levels from individual control (CON; *n* = 17, originating from 4 litters) and poly(I:C)-exposed (POL; *n* = 33, originating from 6 litters) offspring was performed to identify subgroups with different cytokine profiles. **a** Distribution of CON and POL offspring across the two clusters (CL1 and CL2) identified by two-step cluster analysis. The pie charts show the cluster distribution (in percentages, %) for all offspring combined, and for CON and POL offspring separately. The numbers in brackets represent the number of offspring in each cluster. **b** Summary of the relative predictor importance for cluster separation as revealed by two-step cluster analysis. **c** The scatter plots show plasma cytokine levels for subgroups of CON and POL offspring as identified by two-step cluster analysis. Compared to reference CON offspring (Ref-CON, *n* = 16) and POL offspring with a low cytokine status (LCS-POL subgroup, corresponding to POL offspring in CL1; *n* = 20), POL offspring with a high cytokine status (HCS-POL subgroup, corresponding to POL offspring in CL2; *n* = 13) display a significant increase in TNF-α (*F*_(2,46)_ = 19.1, *p* < 0.001; HCS-POL vs. Ref-CON or LCS-POL: ***p < 0.001), IL-1β (*F*_(2,46)_ = 32.6, *p* < 0.001; HCS-POL vs. Ref-CON or LCS-POL: ***p < 0.001), IL-6 (*F*_(2,46)_ = 13.1, *p* < 0.001; HCS-POL vs. Ref-CON or LCS-POL: ***p < 0.001) and IL-10 (*F*_(2,46)_ = 4.9, *p* < 0.05; HCS-POL vs. Ref-CON: **p < 0.01), based on ANOVA and Tukey’s post hoc tests. **d** The scatter plots show behavioral readouts for the same Ref-CON, HCS-POL, and LCS-POL subgroups. Compared to Ref-CON (*n* = 16) and LCS-POL (*n* = 20) subgroups, HCS-POL offspring (*n* = 13) display a significant reduction in the social preference index (*F*_(2,46)_ = 21.1, *p* < 0.001; HCS-POL vs. Ref-CON or LCS-POL: ***p < 0.001) and mean % PPI in the PPI test of the acoustic startle reflex (*F*_(2,46)_ = 10.8, *p* < 0.001; HCS-POL vs. Ref-CON or LCS-POL: **p < 0.01), based on ANOVA and Tukey’s post hoc tests. Spontaneous alternation in the Y-maze working memory test and total distance moved in the open field test were not different between subgroups.

The cluster analysis identified two main clusters (CL1 and CL2) with good cluster separation (silhouette measure of cohesion and separation >0.55). A total of 36 offspring were identified as belonging to CL1, whereas 14 offspring were classified into CL2 (Fig. 5a). The majority of CON offspring (94.1%, 16 out of 17) were assigned to CL1 (Fig. 5a). By contrast, 60.6% (20 out of 33) of the POL offspring were identified as belonging to CL1, whereas the remaining (39.4%; 13 out of 33) were classified into CL2 (Fig. 5a). As shown in Fig. 5b, plasma levels of TNF-α and IL-1β had the highest predictor importance for cluster separation, followed by plasma IL-6 levels. Plasma IFN-γ levels had the lowest predictor importance for cluster separation (Fig. 5b).

In general, offspring classified into CL2 showed a high cytokine status (HCS) in the plasma, whereas those belonging to CL1 had a low cytokine status (LCS). Indeed, compared with the “reference” CON (Ref-CON subgroup, corresponding to CL1-CON offspring) subgroup, HCS-POL offspring (corresponding to CL2-POL offspring) showed increased plasma levels of TNF-α, IL-1β, IL-6, and IL-10, whereas the LCS-POL subgroup (corresponding to CL1-POL offspring) did not differ from Ref-CON offspring (Fig. 5c). These data thus demonstrate that MIA-exposed offspring can be stratified into subgroups displaying distinct plasma cytokine profiles.

Subsequent to the assessment of plasma cytokines and stratification into subgroups, we subjected the same offspring to behavioral testing in order to explore whether HCS-POL and LCS-POL subgroups also differ in their behavioral performances. In support of this hypothesis, we found that HCS-POL offspring displayed a significant reduction in the social preference index and mean % PPI compared to Ref-CON and LCS-POL subgroups (Fig. 5d). There were, however, no differences between the subgroups in terms of spontaneous alternation in the Y-maze working memory test and total distance moved in the open field test (Fig. 5d).

## Discussion

The present study identified and characterized subgroups of isogenic mouse offspring that were exposed to identical MIA induced by gestational administration of the viral mimetic, poly(I:C). Our first strategy, which was based on stratifying the behavioral performance of a large cohort of CON (*n* = 77) and POL (*n* = 81) mice, revealed that ~50% of the MIA-exposed offspring developed overt dysfunctions in behavioral domains relevant to neurodevelopmental disorders, including impairments in social behavior, sensorimotor gating, and working memory, whereas the other half of MIA-exposed offspring was largely indistinguishable from CON offspring in terms of their behavioral performance. This dissociation was similarly seen in male and female offspring and most strongly driven by variations in social approach behavior and PPI of the acoustic startle reflex, suggesting that these two measures can serve as accurate behavioral indices to stratify MIA-exposed offspring into susceptible and resilient subgroups.

The identification of subgroups of MIA-exposed offspring with or without overt behavioral anomalies prompted us to explore molecular and neuroanatomical correlates of resilience and susceptibility to MIA. Our data show that offspring with manifest behavioral impairments (susceptible offspring) differ from asymptomatic (resilient) offspring in terms of their transcriptional profiles in cortical and subcortical brain regions. Notably, even though MIA-exposed offspring of the resilient subgroup did not display apparent behavioral anomalies, they nevertheless showed a number of transcriptional changes in the CNS when compared to nonexposed CON offspring, especially in the Amy. Some of these changes may represent allostatic molecular adaptations to MIA, which in turn may have channeled the developmental trajectories towards behavioral resiliency. Interestingly, the consequences of juvenile stress exposure appear to follow a similar pattern of segregation into susceptible and resilient pathways, with the latter being associated with allostatic adaptations in the central GABAergic system [52]. Our transcriptomic data thus offer the opportunity for future studies to exploit molecular pathways of resilience as novel therapeutic targets for mitigating MIA-associated pathologies.

The presence or absence of behavioral dysfunctions after MIA was, however, not associated with statistically significant subgroup-specific volumetric brain differences as assessed by MRI. Yet, subgroup-specific effects were identified in brain networks of structural covariance [39]. Specifically, for the same regions that were positively correlated to the RSC in the control offspring, reduced structural covariance emerged specifically in susceptible but not resilient offspring of MIA-exposed mothers. These findings suggest that brain connectivity is more profoundly altered in MIA-exposed offspring that show behavioral impairments, as compared to MIA offspring lacking overt behavioral anomalies. Consistent with this view, Krietz et al. [53] provide evidence for abnormal brain functional connectivity in poly(I:C)-exposed mice, as indexed using resting-state functional MRI. Interestingly, the disruption of RSC structural covariance in susceptible MIA-exposed offspring resembles the impairments in RSC connectivity to the anterior cingulate cortex and other default mode network structures in patients with schizophrenia [54] and in individuals with social anhedonia [55]. Collectively, these data suggest that susceptibility and resilience to MIA may not be associated with profound neuroanatomical differences in adult mice, but is characterized by variations in structural covariance, suggestive of abnormal brain connectivity. Although the biological basis of structural covariance is currently unclear, it is linked to patterns of coordinated gene expression [39] and synchronized neurodevelopment [40–42]. Hence, differences in structural covariance may occur as a result of divergent neurodevelopment trajectories in brain structure and function between resilient and susceptible offspring, as already suggested from longitudinal MRI studies of poly(I:C)-exposed rodents that were not stratified into subgroups [33, 34]. Hence, longitudinal multimodal MRI studies in resilient and susceptible MIA-exposed offspring are now warranted to investigate this notion further.

Our second strategy to identify subgroups of MIA-exposed offspring was based on measuring and stratifying inflammation-related cytokines in the plasma of adult CON and POL mice. This approach revealed that ~40% of MIA-exposed offspring displayed elevated production of innate inflammatory cytokines such as IL-1β, IL-6, and TNF-α, whereas the remaining portion of exposed offspring was comparable with controls in terms of plasma cytokine profiles. Long-term changes in inflammatory cytokine secretion have been noted before in rodent and primate models of MIA [48, 50], but these effects appear variable [47, 49]. Consistent with the present data, for example, we previously found that only ~40% of poly(I:C)-exposed mouse offspring develop lasting elevations in inflammatory cytokine production in the ventral midbrain [28]. Similar variability also exists in psychiatric disorders, with schizophrenia being an illustrative example. Indeed, noticeable inflammatory abnormalities in the CNS and/or periphery seem evident only in a subgroup of schizophrenia cases [28, 45, 56–58] and may predict poorer treatment responses and clinical outcomes [43, 59]. In line with the latter notion, we found that the subgroup of MIA-exposed offspring displaying a high inflammatory cytokine status (i.e., the HCS-POL subgroup) also showed significant impairments in social approach behavior and PPI, whereas the subgroup with a low inflammatory cytokine status (i.e., the LCS-POL subgroup) did not. These data suggest that assessing peripheral cytokines may be used to estimate the impact of MIA on offspring behavior, which in turn has relevance for biomarker-guided approaches in preclinical psychiatric research.

By implementing a whole-litter phenotyping approach, we were also able to contrast the contribution of within-litter versus between-litter variability in our model system. Our findings suggest that the stratification of MIA-exposed offspring is, at least in the present model of poly(I:C)-induced MIA, largely driven by within-litter variability. Indeed, in our first experimental series, which included >80 POL offspring from 12 litters, we found that each litter concurrently contained offspring displaying (susceptible subgroup) or lacking (resilient subgroup) behavioral deficits in adulthood. Moreover, in an independent cohort of animals, we revealed that MIA-induced maternal hypothermia, which correlated with the maternal IL-6 response to poly(I:C), did not predict the behavioral outcomes in the adult offspring. The stronger contribution of within-litter variability observed here is consistent with the findings from a recent study using the poly(I:C)-based MIA model in mice, which revealed larger within-litter than between-litter variability in the context of MIA and disruption of cortical interneuron development [60]. Hence, in addition to between-litter variability [6], factors pertaining to within-litter variability should be considered in MIA models that are implemented in litter-bearing mammals such as mice. The factors determining within-litter variability in MIA models remain largely unknown and warrant further investigations. As reviewed elsewhere [22], there are several plausible candidates in this regard, including uteroplacental positioning causing varying immune responses and hormonal exposures during fetal brain development [61, 62], individualization of littermates resulting from the establishment of post-weaning social hierarchies [63, 64], stochastic epigenetic variability and subsequent variation in gene transcription during brain development and maturation [65], and de novo rearrangements caused by retrotransposable elements in the chromosomal DNA of the offspring [66–69].

We acknowledge a number of limitations in our study. First, our experimental approaches to identify and characterize subgroups of MIA-exposed offspring were only conducted once they reached adulthood. Hence, the temporal onset of the dissociation into subgroups remains unknown and should be investigated further using longitudinal studies. Second, while our study identified FDR-corrected differences in the transcriptional profiles of MIA-exposed offspring with (susceptible) or without (resilient) overt behavioral anomalies, the sequencing was based on the use of bulk tissue, which in turn may have masked subtle transcriptional differences occurring in specific neuronal or glial cell populations. Owing to the advances in single-cell biotechnology, the elucidation of cell-type-specific transcriptional alterations may offer valuable insights into the cellular mechanisms underlying the segregation of resilience and susceptibility in the context of MIA. Third, even though we included male and female offspring in the MRI analyses of resilient and susceptible subgroups and controls, these analyses may have been underpowered to detect robust and possibly sex-specific volumetric brain differences across subgroups of MIA-exposed offspring. Finally our study was not designed to ascertain causal relationships between transcriptional and behavioral changes. Exploring such relationships in future studies will be pivotal, as they may open new avenues for novel therapeutic targets against MIA-associated pathologies. Likewise, the extent to which persistent elevations in peripheral cytokines contribute to susceptibility after MIA remains to be determined as well.

In conclusion, our findings identify and characterize phenotypes of resilience and susceptibility emerging after identical MIA in an isogenic mouse line. The continuous identification of factors precipitating phenotypic variability in genetically homogenous populations of MIA-exposed offspring offers the opportunity for future gene–environment interaction studies of MIA-associated pathologies and for exploring the therapeutic or preventive potential of personalized treatments that take these variable effects into consideration. Prenatal poly(I:C) administration in mice and other species is currently one of the most widely used model systems to study immune-mediated developmental brain disorders independently of preexisting diagnostic classifications [20–22]. Therefore, we expect our experimental findings to aid in the characterization of the variable effects MIA may have in the etiology of numerous neurodevelopmental disorders.

## Supplementary information

Supplement 1

Supplement 2

Supplement 3
