# Supplementary material for: Behavioral, neuroanatomical, and molecular correlates of resilience and susceptibility to maternal immune activation
Source: Mol Psychiatry. 2020 Nov 23;26(2):396–410. doi: 10.1038/s41380-020-00952-8 (PMC7850974; doi:10.1038/s41380-020-00952-8)
Supplement: Supplementary file 1 — Supplement 1 [file 41380_2020_952_MOESM1_ESM.pdf]

SUPPLEMENT 1
SUPPLEMENTAL METHODS

Behavioral, neuroanatomical and molecular correlates of resilience and susceptibility to maternal immune activation

Flavia S. Mueller[1], Joseph Scarborough[1], Sina M. Schalbetter[1], Juliet Richetto[1,2], Eugene Kim[3], Amalie Couch[4], Yohan Yee[5], Jason P. Lerch[5,6,7], Anthony C.Vernon[4,8], Ulrike Weber-Stadlbauer[1,2], Urs Meyer[1,2,*]

[1]Institute of Pharmacology and Toxicology, University of Zurich-Vetsuisse, Zurich, Switzerland.
[2]Neuroscience Center Zurich, University of Zurich and ETH Zurich, Zurich, Switzerland.
[3]Department of Neuroimaging, Institute of Psychiatry, Psychology and Neuroscience, King's College London, London, UK.
[4]Department of Basic and Clinical Neuroscience, Institute of Psychiatry, Psychology and Neuroscience, King's College London, London, UK.
[5]Mouse Imaging Centre, Hospital for Sick Children, Toronto, ON, Canada.
[6]Wellcome Centre for Integrative Neuroimaging, University of Oxford, Oxford, UK.
[7]Department of Medical Biophysics, University of Toronto, Toronto, ON, Canada
[8]MRC Centre for Neurodevelopmental Disorders, King's College London, London, UK.

Correspondence:

Urs Meyer, Ph.D.
Institute of Pharmacology and Toxicology
University of Zurich-Vetsuisse
Winterthurerstrasse 260,
8057 Zurich,
Switzerland
E-mail: urs.meyer@vetpharm.uzh.ch
Tel.: +41 44 635 88 44; Fax.: +41 44 635 89 10

***Supplement 1** contains:*
(Named and ordered according to their first mention in the main text)

- Suppl. Table S1
- Suppl. Fig. S6
- Suppl. Table S6

SUPPLEMENTAL METHODS

Animals

C57BL6/N mice were used throughout the whole study. Female and male breeder mice were obtained from Charles River Laboratories (Sulzfeld, Germany) at the age of 10 weeks. Upon arrival, they were housed in individually ventilated cages (IVCs; Allentown Inc., Bussy-Saint-Georges, France) as described in detail before [1]. The cages were kept in a specific-pathogen-free (SPF) holding room, which was temperature- and humidity-controlled (21 ± 3 °C, 50 ± 10%) and kept under a reversed light–dark cycle (lights off: 09:00 AM–09.00 PM). All animals had *ad libitum* access to standard rodent chow (Kliba 3336, Kaiseraugst, Switzerland) and water throughout the entire study. All procedures had been previously approved by the Cantonal Veterinarian's Office of Zurich, Switzerland.

Breeding and maternal manipulations

Independent cohorts of timed-pregnant mice (**Suppl. Table S1**, see below) were generated via on-site breeding, which began two weeks after the animals were acclimatized to our facility. To this end, female and male breeders were subjected to a timed-mating procedure as previously described [1, 2]. Successful mating was verified by the presence of a vaginal plug, upon which dams were housed individually throughout gestation. The presence of a vaginal plug was referred to as gestational day (GD) 0. A dam showing a vaginal plug at GD 0 and a weight gain of ≥ 3 g from GD 0 to GD 12 was considered as undergoing successful pregnancy [2].

On GD 12, pregnant mice were randomly assigned to a single injection of poly(I:C) (potassium salt, P9582, Sigma–Aldrich, Buchs, St Gallen, Switzerland) or treatment with endotoxin-free 0.9% NaCl (B. Braun, Melsungen, Switzerland) vehicle solution. To avoid possible batch-to-batch variation [2], the same batch of poly(I:C) (batch #086M4045) was used in all experiments included in this study. Based on our previous dose-response studies [1, 3], poly(I:C) was administered intravenously (i.v.) into the tail vein at a dose of 5 mg/kg.

The dose of poly(I:C) was calculated based on the pure form and was dissolved in glass vials with vehicle kept at room temperature. Control dams received vehicle solution (i.v.) only. All solutions were freshly prepared on the day of their administration and injected using an injection volume of 5 ml/kg. The tail vein injections were performed under mild physical constraint using a semi-restrictive rodent injection cone (model 561-RC; Plas-Labs Inc., Lansing, USA). Immediately after poly(I:C) or vehicle administration, the dams were placed back to their home cages and left undisturbed until 7 days after birth of the offspring, when the first change of home cage embedding, huts and nesting material was conducted. Offspring of poly(I:C)- or vehicle-treated mothers were weaned on postnatal day (PND) 21, and littermates of the same sex were caged separately and maintained in groups of 3 to 5 animals per cage. Additional methodological details regarding the maternal manipulations are summarized in the reporting guideline checklist for the MIA model [4], as provided in **Supplement 3.**

Supplementary Table S1

Cohort	Number of litters	Number of offspring	Age during testing	Experimental series
**1**	*N*(CON) = 12<br>*N*(POL) = 12	*n*(CON) = 77 (39 m, 38 f)<br>*n*(POL) = 81 (46 m, 35 f)	Adulthood<br>(12 weeks onwards)	**I1**. Behavioral phenotyping and cluster analyses (entire cohort)<br>**I2a.** Next-generation RNA sequencing (3 Ref-CON [m] , 3 Sus-POL [m], 3 Res-POL [m])<br>**I2b.** Ex-vivo MRI (16 Ref-CON [8 m, 8 f], 14 Sus-POL [8 m, 6 f], 14 Res-POL [8 m, 6 f])
**2**	*N*(CON) = 4<br>*N*(POL) = 6	*n*(CON) = 17 (8 m, 9 f)<br>*n*(POL) = 33 (16 m, 17f)	Adulthood<br>(12 weeks onwards)	**II1.** Quantification of cytokines in plasma of adult offspring and cluster analyses (entire cohort)<br>**II2.** Behavioral phenotyping (entire cohort)
**3**	*N*(CON) = 7<br>*N*(POL) = 7	*n*(CON) = 48 (26 m, 22 f)<br>*n*(POL) = 51 (27 m, 24 f)	Adulthood<br>(12-13 weeks)	**III.** Behavioral phenotyping and correlation of behavioral readouts with maternal temperature changes in response to MIA (entire cohort)

Summary of the different cohorts of animals used in this study. Three independent cohorts (cohorts 1-3) of timed-pregnant mice and their offspring were generated via identical on-site breeding. For each cohort, the table summarizes the number of litters (corresponding to the number of treated dams), the number of offspring (per sex), and their allocation to the experimental series of interest. Whereas cohort 1 was used for large-scale behavioral phenotyping and subsequent transcriptional profiling and magnetic resonance imaging (MRI), cohort 2 was used for

measurements of peripheral cytokines, followed by behavioral analyses. Cohort 3 was used to correlate MIA-induced changes in maternal thermoregulation with behavioral profiles in the adult offspring. Note: In cohort 3, an additional sub-cohort of pregnant females (*N*(CON) = 6 and *N*(POL) = 7) was used as satellite animals to correlate poly(I:C)-induced elevation in maternal IL-6 levels with changes in body surface temperature (not shown in the list). CON, vehicle control treatment; m, males; f, females; POL, poly(I:C) treatment.

Behavioral testing

Behavioral testing commenced when the offspring reached 12 weeks of age and included tests for innate anxiety-like behavior and locomotor activity (open field test), working memory (spontaneous alternation in the Y-maze), social interaction (modified version of the three-chamber social interaction test), and PPI of the acoustic startle reflex. These tests were selected because of their relevance to neurodevelopmental disorders [5-7] and because of their wide use in animal models of MIA [4, 8, 9]. All tests were extensively validated previously and are routinely used in our laboratory [1, 3, 10, 11]. Whenever subjected to behavioral testing, each CON and POL offspring was tested repeatedly using the same order of testing (1. open field test; 2. Y-maze working memory test; 3. social interaction test; 4. PPI test), with a resting phase of 3-4 days between individual tests. A detailed description of each behavioral test is provided below:

■ **Open field test.** A standard open field test was used to asses basal locomotor activity and innate anxiety-like behavior in a novel environment [1, 3]. The test was conducted in four identical square arenas (40 × 40 cm) surrounded by walls (35 cm high). The apparatus was made of white Plexiglas and was located in a testing room under lighting (28 lx in the center zone, 20 lx in the corner of the apparatus). A digital camera was mounted directly above the four arenas. Images were captured at a rate of 5 Hz and transmitted to a PC running the Ethovision (Noldus Information Technology, The Netherlands) tracking system. The animals were gently placed in the center of the arena and allowed to freely explore for the duration of 20 min. For the purpose of data collection, the arena was conceptually partitioned into two areas: a center zone (measuring 15 ×15 cm^2) in the middle

of the area, and a peripheral zone occupying the remaining area. The dependent measures were total distance moved (m) in the entire arena, number of center zone visits, time (s) spent in the center zone, and distance moved (m) in the center zone.

■ **Working memory test in the Y-maze.** A spontaneous alternation task in the Y-maze was used to assess working memory. This task is based on the innate tendency of rodents to explore novel environments, that is, their preference to investigate a new arm of the Y-maze rather than returning to one that was previously visited [10]. The apparatus was made of transparent Plexiglas and consisted of three identical arms (50 cm × 9 cm; length × width) surrounded by transparent Plexiglas walls 10 cm in height. The three arms radiated from a central triangle (8 cm on each side) and were spaced 120° from each other. The maze was elevated 90 cm above the floor, and was positioned in a dimly-lit room enriched with distal spatial cues. A digital camera was mounted above the Y-maze apparatus. Images were captured at a rate of 5 Hz, and transmitted to a PC running the EthoVision tracking system (Noldus Information Technology), which calculated the total distance moved (m) and the number of entries into the three arms and the center zone of the Y-maze.

To start the test, the animals were gently placed in the center of the Y maze and allowed to explore freely for 5 min, whereby the number and sequence of arm entries (defined as entry of the whole body into an arm) were observed and recorded by an experimenter who was blinded to the treatment conditions. Alternation was defined as entry into the three arms in any non-repeating order (for example, ABC, BAC, CBA). Working memory was indexed by the percentage alternation score, which was calculated as the total number of alternations divided by the possible alternations given the number of arm entries (total number of arm entries-2). In addition to the analysis of percentage alternation, the total distance moved was recorded and analyzed to assess general activity during the 5-min testing period.

■ **Social interaction test.** Social interaction was assessed by analyzing the relative exploration time between an unfamiliar congenic mouse and an inanimate dummy object using methods established before [1, 12]. The test apparatus was made of Plexiglas and consisted of three identical arms (50 cm × 9 cm; length × width) surrounded by 10-cm high Plexiglas walls. The three arms radiated from a central triangle (8 cm on each side) and spaced 120° from each other. Two out of the three arms contained a rectangular wire grid cage (13 cm × 8 cm × 10 cm, length × width × height; bars horizontally and vertically spaced 9 mm apart). The third arm did not contain a metal wire cage and served as the start zone (see below).

All animals were first habituated to the test apparatus on the day before social interaction testing. This served to familiarize the test animals with the apparatus and to reduce novelty-related locomotor hyperactivity, which may potentially confound social interaction during the critical test phase. The rectangular wire cages (located at the end of two arms) were left empty during the habituation phase. During habituation, each test mouse was gently placed in the start arm and allowed to explore the apparatus for 5 min.

The test phase took place one day after the habituation day. During the test phase, one metal wire cage contained an unfamiliar C57BL6/N mouse of the same sex (10-12 weeks of age), whereas the other wire cage contained an inanimate dummy object. The latter was a black scrunchie made of velvet material. The allocation of the unfamiliar live mouse and inanimate dummy object to the two wire cages was counterbalanced across experimental groups. To start a test trial, the test mouse was gently placed in the start arm and allowed to explore freely for 5 min. Behavioral observations were made by an experimenter who was blinded to the experimental conditions, and social interaction was defined as nose contact within a 2-cm interaction zone. For each animal, a social preference index was calculated the by the formula ([time spent with the mouse] / [time spent with the inanimate object + time spent with the mouse]) - 0.5. The social preference index was used to compare the relative exploration time between the unfamiliar mouse and the inanimate dummy object, with values > 0 signifying a preference towards the unfamiliar mouse. In addition,

the absolute times spent with the unfamiliar mouse and the inanimate dummy object were analyzed. In addition to the analysis of social interaction scores, the total distance moved was recorded and analyzed to assess general activity. This was achieved by a digital camera mounted above the apparatus, which captured images at a rate of 5 Hz. The images were transmitted to a PC running the EthoVision tracking system (Noldus Information Technology), which provided the measure of the total distance moved (m) during the 5-min testing period.

■ **Prepulse inhibition (PPI) of the acoustic startle reflex.** Sensorimotor gating was assessed using the paradigm of prepulse inhibition (PPI) of the acoustic startle reflex. PPI of the acoustic startle reflex refers to the reduction in startle reaction in response to a startle-eliciting pulse stimulus when it is shortly preceded by a weak prepulse stimulus. The apparatus consisted of four startle chambers for mice (San Diego Instruments, San Diego, CA, USA) and has been fully described elsewhere [1, 3]. In the demonstration of PPI, the animals were presented with a series of discrete trials comprising a mixture of four trial types. These included pulse-alone trials, prepulse-plus-pulse trials, prepulse-alone trials, and no-stimulus trials in which no discrete stimulus other than the constant background noise was presented. The pulse and prepulse stimuli used were in the form of a sudden elevation in broadband white noise level (sustaining for 40 and 20 ms, respectively) from the background (65 dB_A), with a rise time of 0.2–1.0 ms. In all trials, three different intensities of pulse (100, 110, and 120 dB_A) and three intensities of prepulse (71, 77, and 83 dB_A, which corresponded to +6, +12, and +18 dB_A above background, respectively) were used. The stimulus-onset asynchrony of the prepulse and pulse stimuli on all prepulse-plus-pulse trials was 100 ms (onset-to-onset).

The protocol used for the PPI test was extensively validated before [1, 12]. A session began with the animals being placed into the Plexiglas enclosure. They were acclimatized to the apparatus for 2 min before the first trial began. The first 6 trials consisted of 6 startle-alone trials; such trials served to habituate and stabilize the animals' startle response and were not included in the analysis. Subsequently, the animals were presented with 10 blocks

of discrete test trials. Each block consisted of the following: three pulse-alone trials (100, 110, or 120 dB_A), 3 prepulse-alone trials (+6, +12, or +18 dB_A above background), 9 possible combinations of prepulse-plus-pulse trials (3 levels of pulse × 3 levels of prepulse), and one no stimulus trial. The 16 discrete trials within each block were presented in a pseudorandom order, with a variable interval of 15 s on average (ranging from 10 to 20 s). For each of the 3 pulse intensities (100, 110, or 120 dB_A), PPI was indexed by percent inhibition of the startle response obtained in the pulse-alone trials by the following expression: 100% × [1 − (mean reactivity on prepulse-plus-pulse trials/mean reactivity on pulse-alone trials)], for each animal, and at each of the three possible prepulse intensities (+6, +12, or +18 dB_A above background). In addition to PPI, reactivity to pulse-alone trials and prepulse-alone trials were also analyzed.

Next-generation RNA sequencing and gene network analysis

We performed next-generation RNA sequencing in the medial prefrontal cortex (mPFC) and amygdala (Amy) in subgroups of behaviorally characterized offspring of cohort 1 (**Suppl. Table S1**, see above) two weeks after behavioral testing. Following decapitation of the animals and subsequent brain extraction, micropunches of mPFC (including anterior cingulate, prelimbic and dorsal parts of the infralimbic cortices; bregma: +2.3 to +1.3 mm) and Amy (including major parts of the basolateral nuclei; bregma: -1.2 to -2.2 mm) regions were prepared as described in detail before [12, 13] and were stored at −80°C until further use.

Total RNA was extracted from mPFC and Amy samples using the SPLIT RNA extraction kit (Lexogen, Austria) following the manufacturer's recommendations and was sent to the Functional Genomics Center in Zurich (FGCZ) for quality control and RNA sequencing. The quality of the RNA was determined with a Fragment Analyzer standard sensitivity RNA measurement (SS RNA kit (15 nt), Agilent, Waldbronn, Germany). The measured concentrations (> 200 ng/µl) and RIN (>8) values qualified for a Poly-A enrichment strategy in order to generate the sequencing libraries applying the TruSeq mRNA Stranded Library Prep Kit (Illumina, Inc, California, USA). After Poly-A selection

using Oligo-dT beads the mRNA was reverse-transcribed into cDNA. The cDNA was fragmented, end-repaired and poly-adenylated before ligation of TruSeq UD Indices (IDT, Coralville, Iowa, USA). The quality and quantity of the amplified sequencing libraries were validated using a Fragment Analyzer SS NGS Fragment Kit (1–6000 bp) (Agilent, Waldbronn, Germany). The equimolar pool of the samples was spiked into a NovaSeq6000 run targeting ~15M reads per sample on a S1 FlowCell (Novaseq S1 Reagent Kit, 100 cycles, Illumina, Inc, California, USA). Reads were quality-checked with FastQC. Sequencing adapters were removed with Trimmomatic [14] and aligned to the reference genome and transcriptome of Mus Musculus (GENCODE, GRCm38,p5) with STAR v2.7.3 [15]. Distribution of the reads across genomic isoform expression was quantified using the R package GenomicRanges from Bioconductor Version 3.10. Minimum mapping quality, as well as minimum feature overlaps, was set to 10. Multi-overlaps were allowed. DEGs were identified using the R package edgeR from Bioconductor Version 3.10, using a generalized linear model (glm) regression, a quasi-likelihood (QL) differential expression test and the trimmed means of M-values (TMM) normalization, thereby applying a FDR correction set at a 5% threshold ($q < 0.05$). Only genes that passed this FDR threshold were considered as significant DEGs.

Functional network prediction was generated through the use of QIAGEN's Ingenuity Pathway Analysis (IPA, QIAGEN Redwood City). IPA uses the curated Ingenuity Knowledge Base to identify the involvement of differentially expressed genes in specific diseases and cellular pathways, and to establish functional networks of direct and indirect interactions between differentially expressed genes based on a functional analysis algorithm [16]. For IPA, we only considered DEGs that passed the FDR threshold ($q < 0.05$) as describe above.

Collection of plasma in pregnant mothers and adult offspring

In cohort 3, pregnant dams were killed by decapitation 3 hrs after vehicle or poly(I:C)-treatment to correlate maternal IL-6 levels with changes in body surface temperature (see

below). Trunk blood was collected into EDTA-containing tubes as described before [2]. The blood was kept on ice for a maximum of 30 min before centrifugation (2000×g, 10 min, 4°C) to collect plasma, which was then stored at −20 °C until further until further use.

In cohort 2, blood was collected from adult offspring one week before behavioral testing (**Suppl. Table S1**, see above). To this aim, mice were subjected to mild physical constraint while the tail was warmed (40°C), followed by a puncture of the lateral tail vein using a 22G needle. The blood was collected into heparinized capillary tubes and kept on ice for a maximum of 30 min before centrifugation (2000×g, 10 min, 4°C) to collect plasma. Plasma was stored at −20 °C until the cytokine assay was performed.

Cytokine measurements

Protein levels of IL-1β, IL-6, IL-10, TNF-α and IFN-γ were quantified in the maternal or offspring's plasma using a customized Meso-Scale Discovery (MSD) V-Plex electrochemoluminescence assay (MSD, Rockville, Maryland, USA) for mice as previously described [1, 2, 17]. The plates were read using the SECTOR PR 400 (MSD) imager and analyzed using MSD's Discovery Workbench analyzer and software package. All assays were run in duplicates according to the manufacturer's instructions. The detection limits were 0.11 pg/ml for IL-1β, 0.63 pg/ml for IL-6, 1.06 pg/ml for IL-10, 0.13 pg/ml for TNF-α, and 0.04 pg/ml for IFN-γ.

Assessment of thermal response

To assess the thermal response to poly(I:C)-induced MIA, we measured maternal surface temperatures at baseline (i.e., immediately before the administration of poly(I:C) or vehicle solution) and 3 h after substance administration. The post-injection interval was chosen because it corresponds to the peak of the hypothermal response to i.v. administration of poly(I:C), which in turn correlates with maternal production of inflammatory cytokines such as IL-6. [2]

The surface temperature was assessed using an infrared camera (Flir E60bx

FLIR®Systems, Emitec, Rotkreuz, Switzerland) using previously validated protocols.[2] In brief, the infrared camera was fixed on a tripod (Kaiser RS 2 XA, Emitec, Rotkreuz, Switzerland) in 35-cm distance from a grid with a marked rectangle (5 cm × 8 cm), in which the mouse was placed. The infrared pictures were analyzed using the software provided by the supplier (FLIR Tools, FLIR®Systems, Emitec, Rotkreuz, Switzerland). To this end, we used the maximal body surface temperature (not including the head and the extremities of the animal) as described before. [2]

Whole brain ex-vivo structural magnetic resonance imaging (MRI)

Two weeks after behavioral testing, a subgroup of behaviorally characterized offspring from cohort 1 (**Suppl. Table S1**, see above) were sacrificed for subsequent whole brain *ex-vivo* structural MR imaging [13]. The animals were deeply anaesthetized with an overdose of Pentobarbital (Streuli Pharma AG, Uznach, Switzerland) and perfused transcardially with 30ml PBS (ThermoFisher Scientific, Zurich, Switzerland) + 1 µl/ml heparin (1000IE/ml, Xtrapharm, Rüti, Switzerland), followed by perfusion with 30ml 4% PFA solution (Artechmis, Zofingen, Switzerland) at a flow rate of 1.0 ml/min. Perfusion-fixed brain tissues were kept intact in the cranium and post-fixed for 24 hours in 4% PFA. The brain samples were then placed in 0.01M phosphate buffer containing 0.05% (w/v) sodium azide to allow tissue re-hydration prior to MRI. Samples were then shipped to Kings College London and stored at 4°C in this solution for 4 weeks prior to *ex vivo* MR image acquisition. Full details of tissue collection, preparation and *ex-vivo* MRI acquisition, image processing and statistical analysis are reported below:

■ **MR image acquisition.** A 9.4T Bruker BioSpec 94/20 horizontal small bore magnet (Bruker Ltd, UK) and a quadrature volume radiofrequency coil (39 mm internal diameter, Rapid Biomedical GmbH, GER) were used for all *ex vivo* MRI acquisitions. Fixed brain samples were placed securely, four at a time, in a custom-made MR-compatible holder and immersed in proton-free susceptibility matching fluid (Fomblin;

Solvay, UK). Samples were scanned in a random order, with the operator blinded to treatment group by numerical coding of samples. Scanning was interspersed with phantoms to ensure consistent operation of the scanner. T2-weighted images were acquired using a 3D fast spin-echo (FSE) sequence with the following parameters: effective echo time (TE) 30 ms, repetition time (TR) 3000 ms, field of view (FOV) 25×25×20 mm and acquisition matrix 250×250×200 yielding isotropic voxels of 100 μm^3. Total scan-time was 5 hours and 44 minutes.

■ **MR image processing.** MR images were visually inspected in native space for artefacts, with no images excluded on this basis. Raw MR Images were converted from the manufacturer's proprietary format to the NIFTI format and processed using a combination of FSL [18], ANTs [19] and the Quantitative Imaging Tools (QUIT) package written in C++ software utilizing the ITK library, available from *https://github.com/spinicist/QUIT.* A template image was constructed from the 3D FSE images of a randomly selected subset of mice using the *antsMultivariateTemplateConstruction2.sh* script with the cross-correlation metric and SyN registration algorithm [20]. The template and individual brains were skull-stripped using the RATS algorithm implemented in QUIT using the *qimask* script [21]. To enable atlas based segmentation (ABS) analysis of regional brain volumes, the study-specific template was then registered to the Allen mouse brain atlas (Common Coordinate Framework version 3 [CCF v3]) using the cross-correlation metric and SyN algorithm in ANTs. Aided by the Allen Institute's anatomical ontology, the 670 regions of interest (ROIs) comprising the CCF v3 were condensed into 71 ROIs with volumes more appropriate to the spatial resolution of our ex vivo MR images (referred to hereafter as DC-MRI atlas). The T2-weighted 3D FSE images for all study subjects were then non-linearly registered to the study template using the *antsRegistrationSyN.sh* script. Jacobian determinant maps of the composite deformation fields from the atlas to the study template to each subject were calculated using the *CreateJacobianDeterminantImage* script (ANTs). The Jacobian determinants in each atlas ROI were summed to calculate ROI

volumes for each subject [22, 23]. The Jacobian determinant maps were log-transformed to allow voxel-wise estimation of apparent volume change via deformation based morphometry (DBM) [24].

■ **MR statistics: Volumetric analysis.** Group level differences in volume (mm^3) were assessed using a combination of ABS and voxel-wise DBM [22, 23, 25]. After image registration and careful checking of atlas label alignment to each individual subject's MR images, we automatically extracted volume volumes for the 71 ROIs comprising the DC-MRI atlas (*see MR image processing*). Total brain volume was calculated from the summation of each individual atlas ROI volumes [22]. All volume data were checked for normality prior to analysis using the Schapiro-Wilk test. Group-level differences in total brain volume (mm^3) were assessed using parametric 1-way analysis of variance (ANOVA). The effects of sex as a biological variable on this measure were also assessed using factorial ANOVA with "sex" as within-subject factor and "group" as between subject factor. Effect sizes for each test were also calculated using either eta squared (η^2) or partial η^2, as appropriate. These analyses were performed using Prism software (v8.4.2; GraphPad, La Jolla, CA, USA) with $\alpha = 0.05$.

Regional volume differences at the group level were assessed for the absolute volumes (mm^3) of all 71 brain ROIs in the DC-MRI atlas using either parametric 1-way ANOVA or non-parametric Kruskal-Wallis 1-way ANOVA as appropriate, with $\alpha = 0.05$ using R-project (v4.0; R Core Team, 2020). The resulting p(ANOVA) values were then corrected for multiple comparisons to account for Type I errors across the 71 individual ROIs using the false-discovery rate (FDR) using Prism software (v8.4.2; GraphPad, La Jolla, CA, USA). A threshold of 5% FDR ($q<0.05$) was considered statistically significant. To calculate the magnitude and direction of volume change for each region between groups, we calculated effect sizes, defined as the difference in means divided by the standard deviation of the comparison group, for example, comparing Ref-CON to Sus-

POL, the effect size = ($\mu_{[Sus\text{-}POL]} - \mu_{[Ref\text{-}CON]})/\sigma_{Ref\text{-}CON}$); measured in units of standard deviation (SD).

For DBM, the voxel-wise analysis of group-level differences in absolute volumes were carried out on the log-transformed Jacobian determinant maps using non-parametric 1-way ANOVA and threshold free cluster enhancement (TFCE) in FSL *randomise* [26]. The resulting F-statistic maps were corrected for multiple comparisons using the family-wise error rate (FWE, $p < 0.05$), and presented in the manuscript at $p < 0.05$ uncorrected for multiple comparisons. In all regional and voxel-wise analyses, the results are presented as sexes combined.

■ **MR statistics: structural covariance.**

Seed definition. We first defined the seed to which structural covariance was assessed by thresholding the TFCE-based F-statistics cluster map associated with absolute volume differences at a threshold of 0.975 and choosing the largest connected component of the resulting binary image.

Target definitions. Projections from the retrosplenial cortex (RSC) emanate to various parts of the brain including the hippocampus, thalamus, pons, and anterior parts of the cortex. Target regions in this analysis are those that are connected to the RSC; we used viral tracing data from the Allen Institute (imaged via serial two-photon microscopy) to create these target ROIs. Briefly, these tracer data result from the injection of a recombinant adeno-associated viral tracer that expresses eGFP into various regions of brains of mice; these tracers label neurons projecting anterograde from the injection sites and do not cross synapses. Injections were performed at P56 +/- 2 days and thus reflect normal structural connectivity in adult mice [27]. In this study, we used viral tracing data pre-processed by the Allen Institute and aligned to their Common Coordinate Framework version 3 (CCFv3) template. These data were the “projection density” image (corresponding to the density of pixels with fluorescent signal in a lower resolution grid)

and “injection fraction” image (proportion of pixels within the manually-defined injection site in a lower resolution grid).

The CCFv3 template on which the viral tracer data are defined were aligned to this study average via ANTs [19, 28], thereby putting the volumetric and tracer data into voxel-to-voxel correspondence (**Suppl. Fig. S6**, see below). We then intersected the injection site labels with the seed region and found that 6 viral tracing experiments were performed such that more than half of the injection site was within our seed region (**Suppl. Table S6**, see below). We merged these tracer images by taking the maximum projection density at each voxel, and subsequently thresholded the merged image to create target masks. Specifically, we binarized this merged tracer image at a projection density of 0.25, and applied the following morphological operations: binary opening to remove thin segments related to signal from axons rather than targets, binary closing to remove holes, and dilation to grow the ROIs.

The subsequent connected components were considered to be targets and labelled; 19 such connected components were identified. Of these 19 components, the largest component arose from high fluorescence at the injection site and overlapped with the seed and was therefore removed. The remaining 18 target ROIs included two that straddled the midline across both hemispheres, and 8 pairs of homotopic regions. Parameters used (e.g. threshold values) in the creation of these target ROIs were chosen before structural covariance was computed.

Structural covariance. Structural covariance is a measure of statistical association between seed and target volumes and can be computed by modelling target volumes as a function of seed volumes and other covariates. In such a modelling framework, group differences in structural covariance can be assessed through an interaction between seed volume and group variables [29]. Here, we extended this framework by using a linear mixed-effects model to simultaneously model this association across all seed-target pairs of regions and subsequently estimate group differences. Mixed-effects

model have been used to assess structural covariance differences before (e.g. [30]). The model we used was:

$$volume_{target} \sim 1 + (1 + group + volume_{seed} + group{:}volume_{seed} \mid target) + group + volume_{seed} + group{:}volume_{seed}$$

In the above model, random effects are conditioned on the target structure. Effects associated with the $volume_{seed}$ term represent structural covariance, and effects associated with the $group{:}volume_{seed}$ interaction term represent structural covariance difference due to group.

This model was fit in R (R Core Team, 2020) using the lmerTest package [31], which calculates p-values based on degrees of freedom estimated from the Satterthwaite approximation. Model fitting relies on the lme4 package [32], using the L-BFGS-B optimizer from the optimx package [33, 34]. Data were further handled with the open-source RMINC (*http://mouse-imaging-centre.github.io/RMINC*) and MRIcrotome (*https://github.com/Mouse-Imaging-Centre/MRIcrotome*) packages. The code is freely available for anyone to reproduce these results.

Supplementary Figure 6

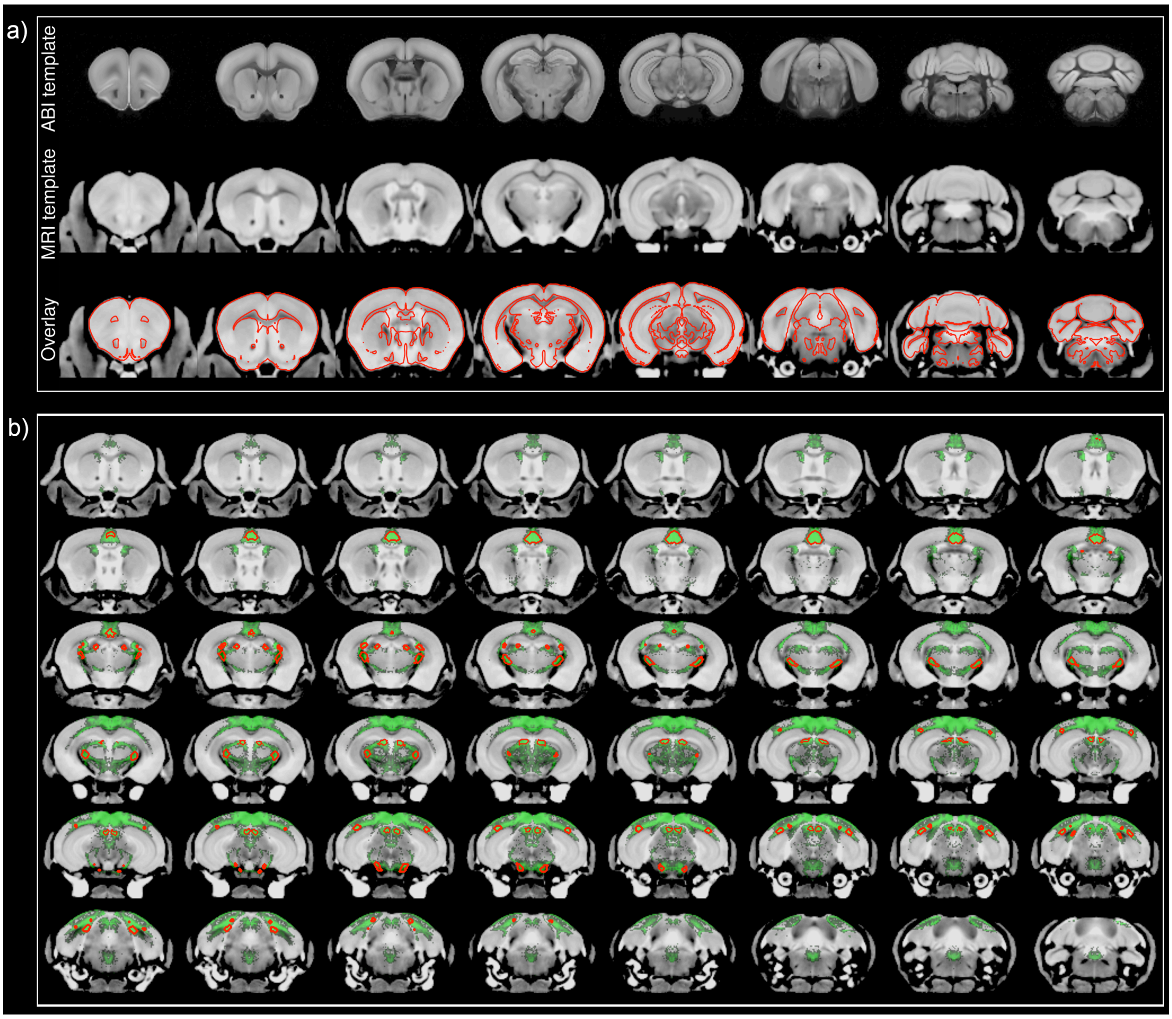

Methods used in determining target regions based on connectivity to retrosplenial cortex (RSC). (a) Alignment between the Allen Institute's CCFv3 template (top row) over which the viral tracing data are defined, and this study's MRI average template, transformed onto the CCFv3 template (middle row). The bottom row shows contours from the CCFv3 template overlaid on the resampled MRI template, to assess registration quality. **(b)** Sequence of coronal slices of the study template, with the merged tracer emanating from the RSC overlaid on top. Contours delineate the 18 target regions defined based on this viral tracing data.

Supplementary Table 6

Experiment	166054929	166054929	538078619	538078619	272735744	272735744
Hemisphere	right	left	right	left	right	left
Seed_size	1682	1682	860	860	579	579
Roi_size	8686	8686	8686	8686	8686	8686
Voxels_seed_covered_by_roi	978	1166	728	812	382	435
Voxels_roi_covered_by_seed	978	1166	728	812	382	435
Percent_of_seed_covered_by_roi	0.581450654	0.693222354	0.846511628	0.944186047	0.659758204	0.751295337
Percent_of_roi_covered_by_seed	0.11259498	0.134239005	0.083813032	0.093483767	0.043978816	0.050080589
Transgenic-line	Rbp4-Cre_KL100	Rbp4-Cre_KL100	Drd3-Cre_KI196	Drd3-Cre_KI196	Cux2-IRES-Cre	Cux2-IRES-Cre
Product-id	5	5	5	5	5	5
Structure-id	886	886	886	886	886	886
Structure-abbrev	RSCv	RSCv	RSCv	RSCv	RSCv	RSCv
Structure-name	Retrosplenial area, ventral part	Retrosplenial area, ventral part	Retrosplenial area, ventral part	Retrosplenial area, ventral part	Retrosplenial area, ventral part	Retrosplenial area, ventral part
Name	Rbp4-Cre-90	Rbp4-Cre-90	Drd3-Cre_KI196-130865	Drd3-Cre_KI196-130865	Cux2-IRES-Cre-275	Cux2-IRES-Cre-275
Injection-volume	0.309425631	0.309425631	0.166729909	0.166729909	0.111492179	0.111492179

Experiment	182467736	182467736	292172100	177907082	177907082	
Hemisphere	right	left	right	right	left	
Seed_size	388	388	173	17	17	
Roi_size	8686	8686	8686	8686	8686	
Voxels_seed_covered_by_roi	337	351	96	17	17	
Voxels_roi_covered_by_seed	337	351	96	17	17	
Percent_of_seed_covered_by_roi	0.868556701	0.904639175	0.554913295	1	1	
Percent_of_roi_covered_by_seed	0.038798066	0.040409855	0.011052268	0.001957172	0.001957172	
Transgenic-line	Cux2-IRES-Cre	Cux2-IRES-Cre	Chrna2-Cre_OE25	Cux2-IRES-Cre	Cux2-IRES-Cre	
Product-id	5	5	5	5	5	
Structure-id	879	879	886	886	886	
Structure-abbrev	RSCd	RSCd	RSCv	RSCv	RSCv	
Structure-name	Retrosplenial area, dorsal part	Retrosplenial area, dorsal part	Retrosplenial area, ventral part	Retrosplenial area, ventral part	Retrosplenial area, ventral part	
Name	Cux2-IRES-Cre-146	Cux2-IRES-Cre-146	Chrna2-Cre_OE25-3899	Cux2-IRES-Cre-130	Cux2-IRES-Cre-130	
Injection-volume	0.081712723	0.081712723	0.033976693	0.004423933	0.004423933	

Metadata related to the selected viral injection experiments from the Allen Institute's Mouse Brain Connectivity Atlas.

Statistical analyses

Since the primary data analysis of our study was based on an unsupervised two-step cluster analysis of a large data set involving > 150 animals (see below), no sample size estimate was calculated to detect a pre-specified effect. Exclusion of animals was not applied.

All statistical analyses of behavioral data and plasma cytokine levels were performed using SPSS Statistics (version 25.0, IBM, Armonk, NY, USA) and Prism (version 8.0; GraphPad Software, La Jolla, California), with statistical significance set at $p < 0.05$. Litter-based analyses of the main behavioral data of cohort 1 (**Suppl. Table S1**, see above), in which the number of litters was considered at the experimental unit, were conducted using independent Student's *t*-tests (two-tailed). *F*-tests for equality of variances were used to compare the dispersion of behavioral data between individual CON and POL offspring from cohort 1. To identify possible subgroups, the main behavioral readouts (total distance moved in the open field, spontaneous alternation in the Y-maze test of working memory, social preference index obtained in the social interaction test, and PPI of the acoustic startle reflex) of individual CON and POL offspring from cohort 1 (**Suppl. Table S1**) were analyzed by two-step cluster analyses [35]. The two-step cluster method uses a pre-clustering step by scanning the entire dataset and storing the dense regions of data records in terms of summary statistics, after which a hierarchical clustering algorithm is then applied to the cluster dense regions [35]. In brief, the main behavioral readouts from each individual CON and POL offspring were fed into the cluster analysis without predetermining the number of clusters, thereby avoiding bias in terms of identifying cluster numbers. The Bayesian information criterion (BIC) was used to estimate the maximum number of clusters, and the log-likelihood method was used as the distance measure [35]. Following stratification of CON and POL offspring into Ref-CON, Sus-POL and Res-POL subgroups by two-step cluster analysis, one-way analysis of variance (ANOVA) and Tukey's post-hoc test for multiple comparisons were used to compare the main behavioral scores between subgroups.

Likewise, two-step cluster analysis was used to identify subgroups of CON and POL offspring with differing plasma cytokine levels (cohort 2, **Suppl. Table S1**). To this end, IL-

1β, IL-6, IL-10, TNF-α and IFN-γ were from individual CON and POL offspring were fed into the cluster analysis without predetermining the number of clusters. Following stratification of CON and POL offspring into POL subgroups with a high cytokine status (HCS-POL) or low cytokine status (LCS-POL), one-way analysis of variance (ANOVA) and Tukey's post-hoc test for multiple comparisons were used to compare the cytokine levels and behavioral scores between HCS-POL and LCS-POL subgroups relative to Ref-CON offspring.

Transcriptomic data were analyzed as described above, using FDR correction set at a 5% threshold ($q < 0.05$). Only genes that passed this FDR threshold were considered as significant DEGs. Neuroanatomical data were analyzed as described above.

Data availability

The FASTQ files associated with the next-generation RNA sequencing of mPFC and Amy samples of Ref-CON, Sus-POL and Res-POL mice have been deposited at the NCBI's Gene Expression Omnibus [36] and are accessible through GEO series accession number GSE150481 (https://www.ncbi.nlm.nih.gov/geo/query/acc.cgi?acc=GSE150481). MR images are freely available on reasonable request to ACV.

Supplemental References

1. Mueller FS, Polesel M, Richetto J, Meyer U, Weber-Stadlbauer U. Mouse models of maternal immune activation: Mind your caging system! *Brain Behav Immun* 2018; **73:** 643-660.

2. Mueller FS, Richetto J, Hayes LN, Zambon A, Pollak DD, Sawa A *et al.* Influence of poly(I:C) variability on thermoregulation, immune responses and pregnancy outcomes in mouse models of maternal immune activation. *Brain Behav Immun* 2019.

3. Meyer U, Feldon J, Schedlowski M, Yee BK. Towards an immuno-precipitated neurodevelopmental animal model of schizophrenia. *Neurosci Biobehav Rev* 2005; **29**(6)**:** 913-947.

4. Kentner AC, Bilbo SD, Brown AS, Hsiao EY, McAllister AK, Meyer U *et al.* Maternal immune activation: reporting guidelines to improve the rigor, reproducibility, and transparency of the model. *Neuropsychopharmacology* 2018.

5. Meyer U, Feldon J, Fatemi SH. In-vivo rodent models for the experimental investigation of prenatal immune activation effects in neurodevelopmental brain disorders. *Neurosci Biobehav Rev* 2009; **33**(7)**:** 1061-1079.

6. Peleg-Raibstein D, Feldon J, Meyer U. Behavioral animal models of antipsychotic drug actions. *Handb Exp Pharmacol* 2012; (212)**:** 361-406.

7. Silverman JL, Yang M, Lord C, Crawley JN. Behavioural phenotyping assays for mouse models of autism. *Nat Rev Neurosci* 2010; **11**(7)**:** 490-502.

8. Brown AS, Meyer U. Maternal Immune Activation and Neuropsychiatric Illness: A Translational Research Perspective. *Am J Psychiatry* 2018**:** appiajp201817121311.

9. Weber-Stadlbauer U, Meyer U. Challenges and opportunities of a-priori and a-posteriori variability in maternal immune activation models. *Current Opinion in Behavioral Sciences* 2019; **28:** 119-128.

10. Willi R, Harmeier A, Giovanoli S, Meyer U. Altered GSK3beta signaling in an infection-based mouse model of developmental neuropsychiatric disease. *Neuropharmacology* 2013; **73:** 56-65.

11. Bitanihirwe BK, Peleg-Raibstein D, Mouttet F, Feldon J, Meyer U. Late prenatal immune activation in mice leads to behavioral and neurochemical abnormalities relevant to the negative symptoms of schizophrenia. *Neuropsychopharmacology* 2010; **35**(12)**:** 2462-2478.

12. Weber-Stadlbauer U, Richetto J, Labouesse MA, Bohacek J, Mansuy IM, Meyer U. Transgenerational transmission and modification of pathological traits induced by prenatal immune activation. *Mol Psychiatry* 2017; **22**(1)**:** 102-112.

13. Richetto J, Chesters R, Cattaneo A, Labouesse MA, Gutierrez AM, Wood TC *et al.* Genome-Wide Transcriptional Profiling and Structural Magnetic Resonance Imaging in the Maternal Immune Activation Model of Neurodevelopmental Disorders. *Cereb Cortex* 2016.

14. Bolger AM, Lohse M, Usadel B. Trimmomatic: a flexible trimmer for Illumina sequence data. *Bioinformatics* 2014; **30**(15)**:** 2114-2120.

15. Dobin A, Davis CA, Schlesinger F, Drenkow J, Zaleski C, Jha S *et al.* STAR: ultrafast universal RNA-seq aligner. *Bioinformatics* 2013; **29**(1)**:** 15-21.

16. Thomas S, Bonchev D. A survey of current software for network analysis in molecular biology. *Hum Genomics* 2010; **4**(5)**:** 353-360.

17. Notter T, Coughlin JM, Gschwind T, Weber-Stadlbauer U, Wang Y, Kassiou M *et al.* Translational evaluation of translocator protein as a marker of neuroinflammation in schizophrenia. *Mol Psychiatry* 2017.

18. Jenkinson M, Beckmann CF, Behrens TE, Woolrich MW, Smith SM. FSL. *Neuroimage* 2012; **62**(2)**:** 782-790.

19. Avants BB, Tustison NJ, Song G, Cook PA, Klein A, Gee JC. A reproducible evaluation of ANTs similarity metric performance in brain image registration. *Neuroimage* 2011; **54**(3)**:** 2033-2044.

20. Avants BB, Yushkevich P, Pluta J, Minkoff D, Korczykowski M, Detre J *et al.* The optimal template effect in hippocampus studies of diseased populations. *Neuroimage* 2010; **49**(3)**:** 2457-2466.

21. Wood TC, Simmons C, Hurley SA, Vernon AC, Torres J, Dell'Acqua F *et al.* Whole-brain ex-vivo quantitative MRI of the cuprizone mouse model. *PeerJ* 2016; **4:** e2632.

22. Doostdar N, Kim E, Grayson B, Harte MK, Neill JC, Vernon AC. Global brain volume reductions in a sub-chronic phencyclidine animal model for schizophrenia and their relationship to recognition memory. *J Psychopharmacol* 2019; **33**(10)**:** 1274-1287.

23. Kuan WL, Stott K, He X, Wood TC, Yang S, Kwok JCF *et al.* Systemic alpha-synuclein injection triggers selective neuronal pathology as seen in patients with Parkinson's disease. *Mol Psychiatry* 2019.

24. Richetto J, Massart R, Weber-Stadlbauer U, Szyf M, Riva MA, Meyer U. Genome-wide DNA Methylation Changes in a Mouse Model of Infection-Mediated Neurodevelopmental Disorders. *Biol Psychiatry* 2017; **81**(3)**:** 265-276.

25. Crum WR, Sawiak SJ, Chege W, Cooper JD, Williams SCR, Vernon AC. Evolution of structural abnormalities in the rat brain following in utero exposure to maternal immune activation: A longitudinal in vivo MRI study. *Brain Behav Immun* 2017; **63:** 50-59.

26. Smith SM, Nichols TE. Threshold-free cluster enhancement: addressing problems of smoothing, threshold dependence and localisation in cluster inference. *Neuroimage* 2009; **44**(1)**:** 83-98.

27. Oh SW, Harris JA, Ng L, Winslow B, Cain N, Mihalas S *et al.* A mesoscale connectome of the mouse brain. *Nature* 2014; **508**(7495)**:** 207-214.

28. Avants B, Duda JT, Kim J, Zhang H, Pluta J, Gee JC *et al.* Multivariate analysis of structural and diffusion imaging in traumatic brain injury. *Acad Radiol* 2008; **15**(11)**:** 1360-1375.

29. Lerch JP, Worsley K, Shaw WP, Greenstein DK, Lenroot RK, Giedd J *et al.* Mapping anatomical correlations across cerebral cortex (MACACC) using cortical thickness from MRI. *Neuroimage* 2006; **31**(3)**:** 993-1003.

30. Khundrakpam BS, Lewis JD, Reid A, Karama S, Zhao L, Chouinard-Decorte F *et al.* Imaging structural covariance in the development of intelligence. *Neuroimage* 2017; **144**(Pt A)**:** 227-240.

31. Kuznetsova A, Brockhoff PB, Christensen RHB. lmerTest Package: Tests in Linear Mixed Effects Models. *Journal of Statistical Software; Vol 1, Issue 13 (2017)* 2017.

32. Bates D, Mächler M, Bolker B, Walker S. Fitting Linear Mixed-Effects Models Using lme4. *Journal of Statistical Software; Vol 1, Issue 1 (2015)* 2015.

33. Nash JC, Varadhan R. Unifying Optimization Algorithms to Aid Software System Users: optimx for R. *Journal of Statistical Software; Vol 1, Issue 9 (2011)* 2011.

34. Nash JC. On Best Practice Optimization Methods in R. *Journal of Statistical Software; Vol 1, Issue 2 (2014)* 2014.

35. Purves-Tyson TD, Weber-Stadlbauer U, Richetto J, Rothmond DA, Labouesse MA, Polesel M *et al.* Increased levels of midbrain immune-related transcripts in schizophrenia and in murine offspring after maternal immune activation. *Mol Psychiatry* 2019.

36. Edgar R, Domrachev M, Lash AE. Gene Expression Omnibus: NCBI gene expression and hybridization array data repository. *Nucleic Acids Res* 2002; **30**(1)**:** 207-210.
