# Supplementary material for: Behavioral, neuroanatomical, and molecular correlates of resilience and susceptibility to maternal immune activation
Source: Mol Psychiatry. 2020 Nov 23;26(2):396–410. doi: 10.1038/s41380-020-00952-8 (PMC7850974; doi:10.1038/s41380-020-00952-8)
Supplement: Supplementary file 2 — Supplement 2 [file 41380_2020_952_MOESM2_ESM.pdf]

SUPPLEMENT 2
SUPPLEMENTAL RESULTS

Behavioral, neuroanatomical and molecular correlates of resilience and susceptibility to maternal immune activation

Flavia S. Mueller[1], Joseph Scarborough[1], Sina M. Schalbetter[1], Juliet Richetto[1,2], Eugene Kim[3], Amalie Couch[4], Yohan Yee[5], Jason P. Lerch[5,6,7], Anthony C.Vernon[4,8], Ulrike Weber-Stadlbauer[1,2], Urs Meyer[1,2,*]

[1]Institute of Pharmacology and Toxicology, University of Zurich-Vetsuisse, Zurich, Switzerland.
[2]Neuroscience Center Zurich, University of Zurich and ETH Zurich, Zurich, Switzerland.
[3]Department of Neuroimaging, Institute of Psychiatry, Psychology and Neuroscience, King's College London, London, UK.
[4]Department of Basic and Clinical Neuroscience, Institute of Psychiatry, Psychology and Neuroscience, King's College London, London, UK.
[5]Mouse Imaging Centre, Hospital for Sick Children, Toronto, ON, Canada.
[6]Wellcome Centre for Integrative Neuroimaging, University of Oxford, Oxford, UK.
[7]Department of Medical Biophysics, University of Toronto, Toronto, ON, Canada
[8]MRC Centre for Neurodevelopmental Disorders, King's College London, London, UK.

Correspondence:

Urs Meyer, Ph.D.
Institute of Pharmacology and Toxicology
University of Zurich-Vetsuisse
Winterthurerstrasse 260,
8057 Zurich,
Switzerland
E-mail: urs.meyer@vetpharm.uzh.ch
Tel.: +41 44 635 88 44; Fax.: +41 44 635 89 10

***Supplement 2** contains:*
(Named and ordered according to their first mention in the main text)

- Suppl. Fig. S1
- Suppl. Fig. S2
- Suppl. Fig. S3
- Suppl. Table S2
- Suppl. Table S3
- Suppl. Table S4
- Suppl. Fig. S4
- Suppl. Fig. S5
- Suppl. Table S5
- Suppl. Fig. S7

SUPPLEMENTAL FIGURES AND TABLES

(Ordered according to their first mention in the main text)

Supplemental Figure S1

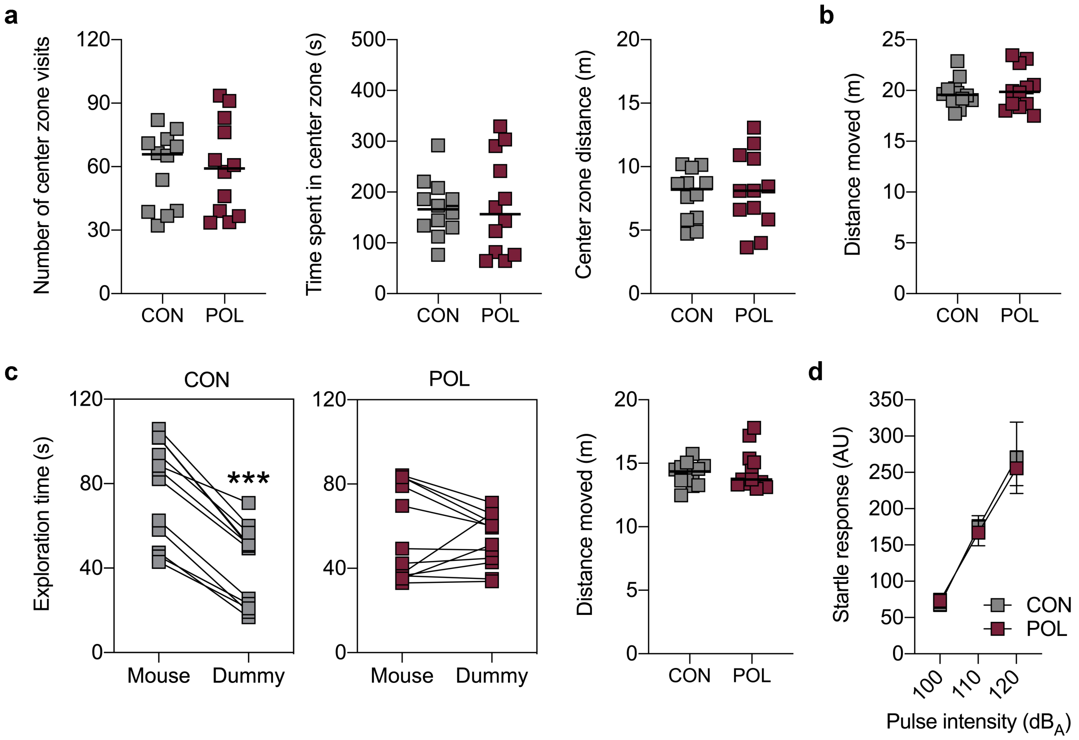

Secondary behavioral read-outs of MIA-exposed and control offspring of cohort 1. Pregnant C57BL6/N mice were exposed to poly(I:C) (POL) or control (CON) treatment on gestation day 12. At adult age (12 weeks onwards), the resulting offspring were subjected to behavioral testing in the open field task, Y-maze spontaneous alternation task, social interaction task, and prepulse inhibition (PPI) task. The secondary behavioral read-outs shown here correspond to litter-based analyses, in which the number of litters (*N* = 12 per treatment group) was considered as experimental unit, and relate to the data depicted in ***Figure 1*** of the main text. **(a)** Number of center zone visits, time (s) spent in the center zone, and distance moved (m) in the center zone in the open field test. **(b)** Total distance moved in the Y-maze spontaneous alternation task of working memory. **(c)** Absolute exploration times (s) of an unfamiliar mouse (mouse) and inanimate dummy object (dummy), and total distance moved (m) in the social interaction test. ***$p < 0.001$ (two-tailed *t*-test), reflecting the significant difference between mouse and dummy exploration times in CON offspring. **(D)** Pulse-induced startle reactivity (in arbitrary units, AU) as a function of pulse intensity (100, 110 and 120 dB_A).

Supplemental Figure S2

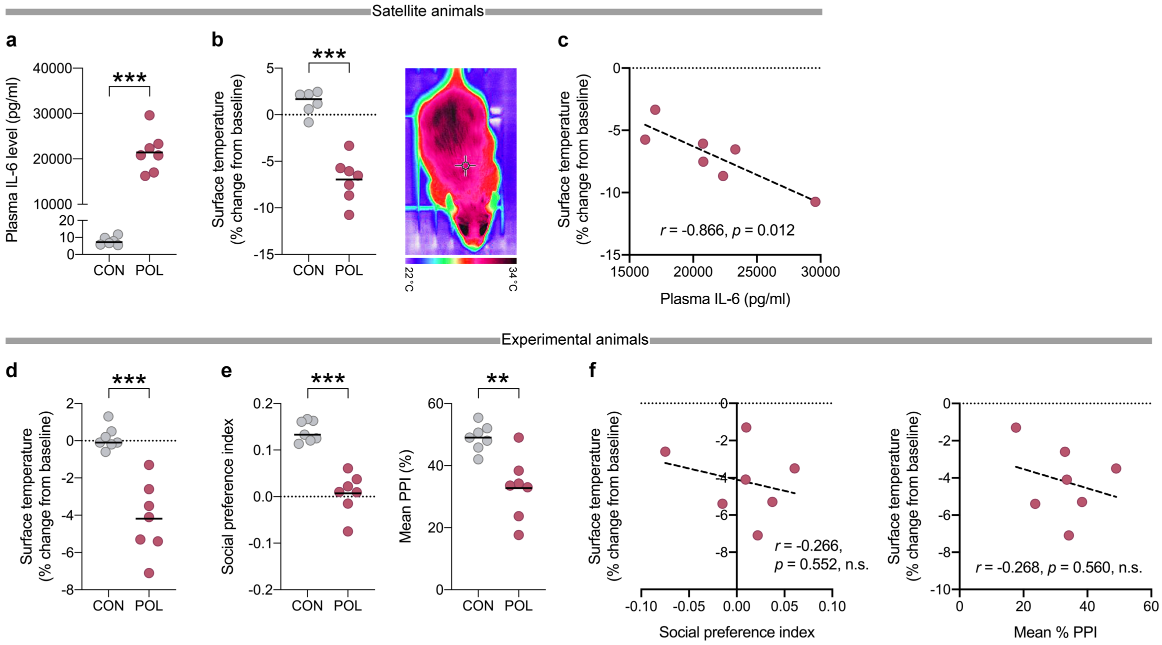

Relationship between MIA-induced changes in maternal IL-6 levels, thermoregulation and offspring behavior. Pregnant C57BL6/N mice were exposed to poly(I:C) (POL) or control (CON) treatment on gestation day 12. Animals in (a-c) represent pregnant females (*N*(CON) = 6 and *N*(POL) = 7) was used as satellite animals to correlate poly(I:C)-induced elevation in maternal IL-6 levels with changes in body surface temperature, whereas the experimental animals in (d-f) represent pregnant females (*N*(CON) = 7 and *N*(POL) = 7) and their offspring (*n*(CON) = 48 (26 m, 22 f), *n*(POL) = 51 (27 m, 24 f); from 7 litters each) of cohort 3 (see ***Suppl. Table S1***) , which were used to correlate MIA-induced changes in maternal thermoregulation with behavioral profiles in the adult offspring. **(a)** Maternal plasma levels of IL-6 protein 3 hrs after CON or POL treatment in satellite animals. ***$p < 0.001$, based on two-tailed t-test ($t_{(11)} = 11.74$). **(b)** Maternal body surface temperature (expressed as % change from baseline temperature) 3 hrs after CON or POL treatment in satellite animals. ***$p < 0.001$, based on two-tailed t-test ($t_{(11)} = 7.67$). **(c)** Bivariate Pearson's correlation between maternal body surface temperature and plasma Il-6 levels 3 hrs after POL treatment in satellite animals. **(d)** Maternal body surface temperature (expressed as % change from baseline temperature) 3 hrs after CON or POL treatment in experimental animals of cohort 3. ***$p < 0.001$, based on two-tailed t-test ($t_{(12)} = 5.64$). **(e)** Litter-based analyses of social preference index and mean percent prepulse inhibition (mean % PPI) in adult offspring of CON- or POL-treated mothers of cohort 3, in which the number of litters (*N* = 7 per treatment group) was considered as experimental unit. **$p < 0.01$ ($t_{(12)} = 3.19$) and ***$p < 0.001$ ($t_{(12)} = 7.25$), based on two-tailed t-tests. **(f)** Bivariate Pearson's correlations between maternal body surface temperature and behavioral profiles (social preference index and mean % PPI) of adult POL offspring of cohort 3.

Supplemental Figure S3

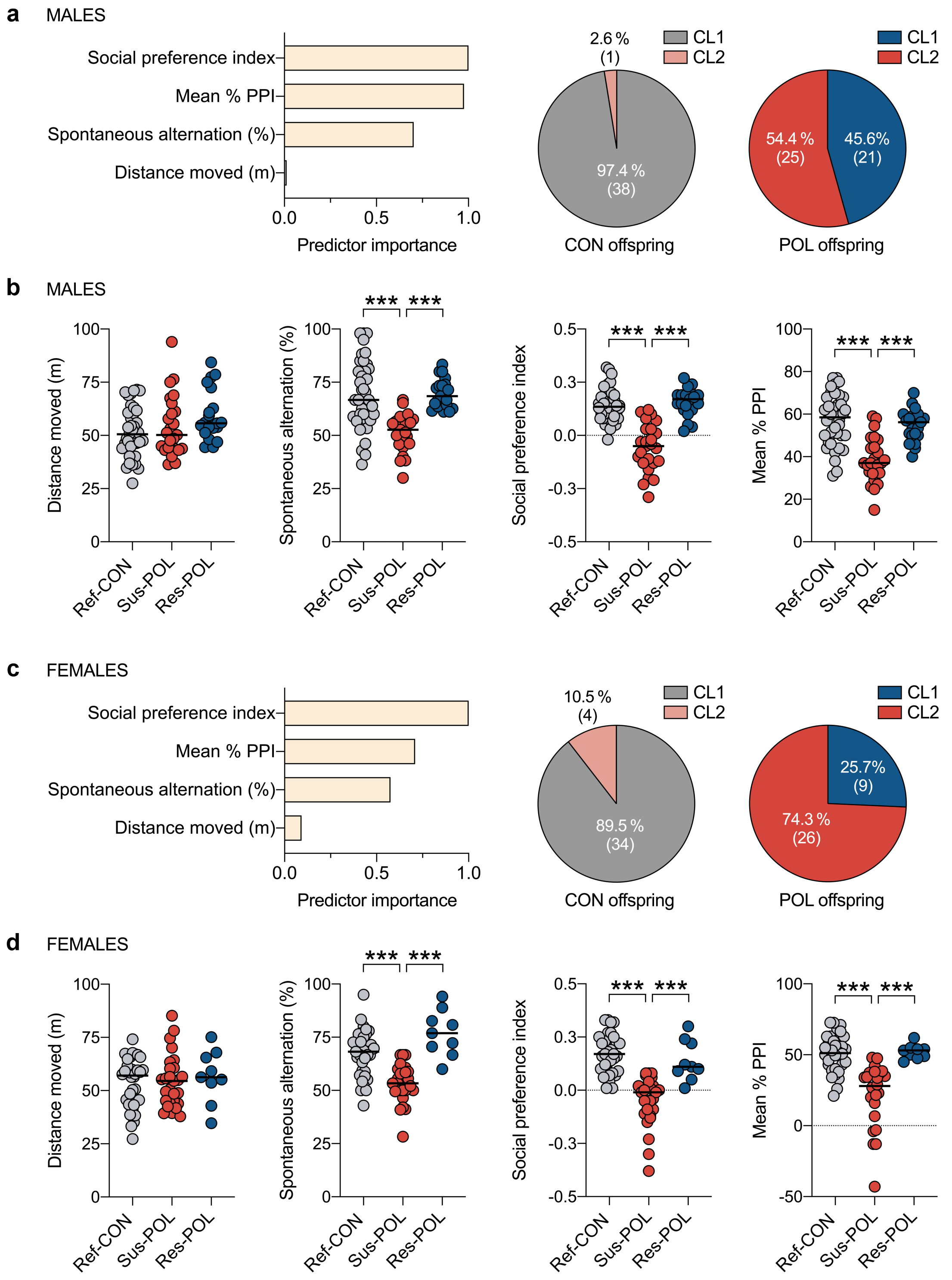

Stratification of MIA-exposed offspring into resilient and susceptible subgroups separated by sex. For either sex, a two-step cluster analysis imputing the main behavioral measures (total distance moved in the open field, spontaneous alternation in the Y-maze test of working memory, social preference index in the social interaction test, and mean % PPI of the acoustic startle reflex) from individual control (CON; n = 39 males and n = 38 females) and poly(I:C)-exposed (POL; n = 46 males and n = 35 females) offspring was performed to identify subgroups with differing

behavioral profiles. The data shown here stem from offspring of cohort 1 and relate to the data depicted in ***Figure 2*** of the main text. **(a)** Summary of the relative predictor importance for cluster separation and distribution (in percentage, %) of male CON and POL offspring across the two clusters (CL1 and CL2) identified by two-step cluster analysis. The numbers in brackets represent the number of male offspring in each cluster. **(b)** The scatter plots show the main behavioral readouts (distance moved in the open field test, spontaneous alternation in the Y-maze working memory test, social preference index in the social interaction test, and mean % PPI in the PPI test of the acoustic startle reflex) for distinct subgroups of male CON and POL offspring as identified by two-step cluster analysis. Resilient POL (Res-POL, n = 21) and susceptible POL (Sus-POL, n = 25) male offspring represent offspring belonging to CL1 and CL2, respectively, whereas reference CON (Ref-CON, n = 38) male offspring were identified as belonging to CL1. ***p < 0.001, based on Tukey's post-hoc tests following one-way ANOVA. **(c)** Summary of the relative predictor importance for cluster separation and distribution (in percentage, %) of female CON and POL offspring across the two clusters (CL1 and CL2) identified by two-step cluster analysis. The numbers in brackets represent the number of female offspring in each cluster. **(d)** The scatter plots show the main behavioral readouts (distance moved in the open field test, spontaneous alternation in the Y-maze working memory test, social preference index in the social interaction test, and mean % PPI in the PPI test of the acoustic startle reflex) for distinct subgroups of female CON and POL offspring as identified by two-step cluster analysis. Resilient POL (Res-POL, n = 9) and susceptible POL (Sus-POL, n = 26) female offspring represent offspring belonging to CL1 and CL2, respectively, whereas reference CON (Ref-CON, n = 34) female offspring were identified as belonging to CL1. ***p < 0.001, based on Tukey's post-hoc tests following one-way ANOVA.

Supplemental Table S2

			mPFC				Amy			
			Sus-POL over Ref-CON		Res-POL over Ref-CON		Sus-POL over Ref-CON		Res-POL over Ref-CON	
**Pathway**	**Gene symbol**	**Gene name**	**Log2FC**	***q*-value**	**Log2FC**	***q*-value**	**Log2FC**	***q*-value**	**Log2FC**	***q*-value**
**Opioid signaling**	*ADCY5*	adenylate cyclase 5	N/A	N/A	N/A	N/A	0.546	0.028	N/A	N/A
	AKT2	AKT serinethreonine kinase 2	N/A	N/A	N/A	N/A	N/A	N/A	0.428	0.029
	AP2S1	adaptor-related protein complex 2, sigma 1 subunit	-0.595	0.046	N/A	N/A	N/A	N/A	N/A	N/A
	CACNA1H	calcium channel, voltage-dependent, T type, alpha 1H subunit	-0.687	0.046	N/A	N/A	N/A	N/A	N/A	N/A
	CACNA1I	calcium voltage-gated channel subunit alpha1 I	N/A	N/A	N/A	N/A	N/A	N/A	0.554	0.020
	CACNG3	calcium voltage-gated channel auxiliary subunit gamma 3	N/A	N/A	N/A	N/A	0.449	0.028	N/A	N/A
	CACNG4	calcium voltage-gated channel auxiliary subunit gamma 4	N/A	N/A	N/A	N/A	0.519	0.023	N/A	N/A
	CACNG7	calcium channel, voltage-dependent, gamma subunit 7	-0.523	0.047	N/A	N/A	N/A	N/A	N/A	N/A

	CACNG8	calcium channel, voltage-dependent, gamma subunit 8	-0.648	0.043	N/A	N/A	N/A	N/A	N/A	N/A
	CAMK2G	calciumcalmodulin dependent protein kinase II gamma	N/A	N/A	N/A	N/A	N/A	N/A	0.421	0.043
	EGR4	early growth response 4	-1.586	0.020	-1.0000	0.0437	N/A	N/A	N/A	N/A
	GNAI2	guanine nucleotide binding protein (G protein), alpha inhibiting 2	-0.399	0.045	N/A	N/A	N/A	N/A	N/A	N/A
	GNAL	G protein subunit alpha L	N/A	N/A	N/A	N/A	0.497	0.017	N/A	N/A
	GNG7	G protein subunit gamma 7	N/A	N/A	N/A	N/A	0.566	0.022	N/A	N/A
	GRIN2B	glutamate receptor, ionotropic, NMDA2B (epsilon 2)	0.457	0.046	N/A	N/A	N/A	N/A	N/A	N/A
	GRIN3A	glutamate ionotropic receptor NMDA type subunit 3A	N/A	N/A	N/A	N/A	N/A	N/A	-0.441	0.023
	ITPR1	inositol 1,4,5-trisphosphate receptor type 1	N/A	N/A	N/A	N/A	N/A	N/A	0.518	0.036
	MAP2K2	mitogen-activated protein kinase kinase 2	-0.704	0.040	N/A	N/A	N/A	N/A	N/A	N/A
	MAP2K7	mitogen-activated protein kinase kinase 7	-0.455	0.043	N/A	N/A	N/A	N/A	N/A	N/A
	MAPK4	mitogen-activated protein kinase 4	N/A	N/A	N/A	N/A	0.533	0.022	N/A	N/A
	MAPK6	mitogen-activated protein kinase 6	N/A	N/A	N/A	N/A	N/A	N/A	0.560	0.021
	MRAS	muscle RAS oncogene homolog	N/A	N/A	N/A	N/A	0.359	0.049	N/A	N/A
	PDE1B	phosphodiesterase 1B	N/A	N/A	N/A	N/A	0.586	0.026	N/A	N/A
	PDYN	prodynorphin	N/A	N/A	N/A	N/A	0.654	0.027	0.925	0.020
	PENK	proenkephalin	N/A	N/A	N/A	N/A	0.565	0.021	N/A	N/A
	PRKCA	protein kinase C alpha	N/A	N/A	N/A	N/A	N/A	N/A	-0.696	0.015
	PRKCG	protein kinase C gamma	N/A	N/A	N/A	N/A	N/A	N/A	-0.636	0.014
	RASD1	ras related dexamethasone induced 1	N/A	N/A	N/A	N/A	-0.914	0.021	-1.400	0.006
	RASD2	RASD family member 2	N/A	N/A	N/A	N/A	0.861	0.021	1.247	0.049
	RGS17	regulator of G-protein signaling 17	0.476	0.039	N/A	N/A	N/A	N/A	N/A	N/A
	RGS4	regulator of G protein signaling 4	N/A	N/A	N/A	N/A	N/A	N/A	0.853	0.014
	RGS6	regulator of G protein signaling 6	N/A	N/A	N/A	N/A	0.597	0.044	0.670	0.041
	RGS8	regulator of G protein signaling 8	N/A	N/A	N/A	N/A	0.394	0.036	N/A	N/A
	RPS6KA3	ribosomal protein S6 kinase A3	N/A	N/A	N/A	N/A	N/A	N/A	0.470	0.044
	RPS6KB1	ribosomal protein S6 kinase, polypeptide 1	0.391	0.046	N/A	N/A	N/A	N/A	N/A	N/A
**G-protein-coupled receptor signaling**	*ADCY5*	adenylate cyclase 5	N/A	N/A	N/A	N/A	0.546	0.028	N/A	N/A
	ADORA2A	adenosine A2a receptor	N/A	N/A	N/A	N/A	0.759	0.019	N/A	N/A
	ADRA2C	adrenergic receptor, alpha 2c	-0.658	0.040	N/A	N/A	N/A	N/A	N/A	N/A
	AKT2	AKT serinethreonine kinase 2	N/A	N/A	N/A	N/A	N/A	N/A	0.428	0.029
	CAMK2G	calciumcalmodulin dependent protein kinase II gamma	N/A	N/A	N/A	N/A	N/A	N/A	0.421	0.043
	CHRM4	cholinergic receptor muscarinic 4	N/A	N/A	N/A	N/A	N/A	N/A	0.852	0.045
	CHRM5	cholinergic receptor muscarinic 5	N/A	N/A	N/A	N/A	-1.185	0.022	-1.408	0.027
	DRD1	dopamine receptor D1	N/A	N/A	N/A	N/A	0.724	0.020	N/A	N/A
	DRD5	dopamine receptor D5	N/A	N/A	N/A	N/A	N/A	N/A	-1.117	0.033
	DUSP1	dual specificity phosphatase 1	N/A	N/A	N/A	N/A	N/A	N/A	0.926	0.022

	GNAI2	guanine nucleotide binding protein (G protein), alpha inhibiting 2	-0.399	0.045	N/A	N/A	N/A	N/A	N/A	N/A
	GNAL	G protein subunit alpha L	N/A	N/A	N/A	N/A	0.497	0.017	N/A	N/A
	GRM4	glutamate receptor, metabotropic 4	-1.066	0.041	N/A	N/A	N/A	N/A	N/A	N/A
	HTR1A	5-hydroxytryptamine receptor 1A	N/A	N/A	N/A	N/A	-0.584	0.043	-1.048	0.002
	HTR2C	5-hydroxytryptamine receptor 2C	N/A	N/A	N/A	N/A	N/A	N/A	-1.056	0.004
	Htr5b	5-hydroxytryptamine (serotonin) receptor 5B	N/A	N/A	N/A	N/A	N/A	N/A	-1.445	0.016
	MAP2K2	mitogen-activated protein kinase kinase 2	-0.704	0.040	N/A	N/A	N/A	N/A	N/A	N/A
	MRAS	muscle RAS oncogene homolog	N/A	N/A	N/A	N/A	0.359	0.049	N/A	N/A
	NFKBIB	nuclear factor of kappa light polypeptide gene enhancer in B cells inhibitor, beta	-0.850	0.031	N/A	N/A	N/A	N/A	N/A	N/A
	PDE10A	phosphodiesterase 10A	N/A	N/A	N/A	N/A	0.916	0.022	N/A	N/A
	PDE11A	phosphodiesterase 11A	N/A	N/A	N/A	N/A	N/A	N/A	-1.205	0.040
	PDE1B	phosphodiesterase 1B	N/A	N/A	N/A	N/A	0.586	0.026	N/A	N/A
	PDE7B	phosphodiesterase 7B	N/A	N/A	N/A	N/A	0.703	0.010	1.038	0.025
	PDE8B	phosphodiesterase 8B	N/A	N/A	N/A	N/A	N/A	N/A	0.530	0.018
	PIK3C2B	phosphatidylinositol-4-phosphate 3-kinase catalytic subunit type 2 beta	N/A	N/A	N/A	N/A	N/A	N/A	0.820	0.013
	PIK3R2	phosphoinositide-3-kinase regulatory subunit 2	-0.747	0.034	N/A	N/A	N/A	N/A	N/A	N/A
	PLCB4	phospholipase C beta 4	N/A	N/A	N/A	N/A	N/A	N/A	0.769	0.021
	PRKCA	protein kinase C alpha	N/A	N/A	N/A	N/A	N/A	N/A	-0.696	0.015
	PRKCG	protein kinase C gamma	N/A	N/A	N/A	N/A	N/A	N/A	-0.636	0.014
	RASD1	ras related dexamethasone induced 1	N/A	N/A	N/A	N/A	-0.914	0.021	-1.400	0.006
	RASD2	RASD family member 2	N/A	N/A	N/A	N/A	0.861	0.021	1.247	0.049
	RGS4	regulator of G protein signaling 4	N/A	N/A	N/A	N/A	N/A	N/A	0.853	0.014
**CXCR4 signaling**	*ADCY5*	adenylate cyclase 5	N/A	N/A	N/A	N/A	0.546	0.028	N/A	N/A
	AKT2	AKT serinethreonine kinase 2	N/A	N/A	N/A	N/A	N/A	N/A	0.428	0.029
	BCAR1	BCAR1 scaffold protein, Cas family member	-0.627	0.026	N/A	N/A	N/A	N/A	N/A	N/A
	CD4	CD4 molecule	N/A	N/A	N/A	N/A	0.863	0.049	N/A	N/A
	EGR1	early growth response 1	N/A	N/A	N/A	N/A	0.662	0.028	1.198	0.010
	GNA12	G protein subunit alpha 12	-0.625	0.026	N/A	N/A	N/A	N/A	N/A	N/A
	GNAI2	G protein subunit alpha i2	-0.399	0.045	N/A	N/A	N/A	N/A	N/A	N/A
	GNAL	G protein subunit alpha L	N/A	N/A	N/A	N/A	0.497	0.017	N/A	N/A
	GNB2	G protein subunit beta 2	-0.631	0.038	N/A	N/A	N/A	N/A	N/A	N/A
	GNG7	G protein subunit gamma 7	N/A	N/A	N/A	N/A	0.566	0.022	N/A	N/A
	ITPR1	inositol 1,4,5-trisphosphate receptor type 1	N/A	N/A	N/A	N/A	N/A	N/A	0.518	0.036
	JUN	Jun proto-oncogene, AP-1 transcription factor subunit	-0.801	0.046	N/A	N/A	N/A	N/A	N/A	N/A
	MAP2K2	mitogen-activated protein kinase kinase 2	-0.704	0.040	N/A	N/A	N/A	N/A	N/A	N/A
	MRAS	muscle RAS oncogene homolog	N/A	N/A	N/A	N/A	0.359	0.049	N/A	N/A
	MYL4	myosin light chain 4	N/A	N/A	N/A	N/A	0.671	0.035	N/A	N/A

	PAK3	p21 (RAC1) activated kinase 3	0.497	0.040	N/A	N/A	N/A	N/A	N/A	N/A
	PIK3C2B	phosphatidylinositol-4-phosphate 3-kinase catalytic subunit type 2 beta	N/A	N/A	N/A	N/A	N/A	N/A	0.820	0.013
	PIK3R2	phosphoinositide-3-kinase regulatory subunit 2	-0.747	0.034	N/A	N/A	N/A	N/A	N/A	N/A
	PLCB4	phospholipase C beta 4	N/A	N/A	N/A	N/A	N/A	N/A	0.769	0.021
	PRKCA	protein kinase C alpha	N/A	N/A	N/A	N/A	N/A	N/A	-0.696	0.015
	PRKCG	protein kinase C gamma	N/A	N/A	N/A	N/A	N/A	N/A	-0.636	0.014
	RASD1	ras related dexamethasone induced 1	N/A	N/A	N/A	N/A	-0.914	0.021	-1.400	0.006
	RASD2	RASD family member 2	N/A	N/A	N/A	N/A	0.861	0.021	1.247	0.049
	RHOB	ras homolog family member B	-0.544	0.031	N/A	N/A	N/A	N/A	N/A	N/A
	RHOBTB2	Rho related BTB domain containing 2	N/A	N/A	N/A	N/A	0.530	0.014	0.585	0.009
	RHOG	ras homolog family member G	-0.678	0.039	-0.9144	0.0437	N/A	N/A	N/A	N/A
**CREB signaling**	*ADCY5*	adenylate cyclase 5	N/A	N/A	N/A	N/A	0.546	0.028	N/A	N/A
	AKT2	AKT serinethreonine kinase 2	N/A	N/A	N/A	N/A	N/A	N/A	0.428	0.029
	CACNA1H	calcium voltage-gated channel subunit alpha1 H	-0.687	0.046	N/A	N/A	N/A	N/A	N/A	N/A
	CACNA1I	calcium voltage-gated channel subunit alpha1 I	N/A	N/A	N/A	N/A	N/A	N/A	0.554	0.020
	CACNG3	calcium voltage-gated channel auxiliary subunit gamma 3	N/A	N/A	N/A	N/A	0.449	0.028	N/A	N/A
	CACNG4	calcium voltage-gated channel auxiliary subunit gamma 4	N/A	N/A	N/A	N/A	0.519	0.023	N/A	N/A
	CACNG7	calcium voltage-gated channel auxiliary subunit gamma 7	-0.523	0.047	N/A	N/A	N/A	N/A	N/A	N/A
	CACNG8	calcium voltage-gated channel auxiliary subunit gamma 8	-0.648	0.043	N/A	N/A	N/A	N/A	N/A	N/A
	CAMK2G	calciumcalmodulin dependent protein kinase II gamma	N/A	N/A	N/A	N/A	N/A	N/A	0.421	0.043
	GNA12	G protein subunit alpha 12	-0.625	0.026	N/A	N/A	N/A	N/A	N/A	N/A
	GNAI2	G protein subunit alpha i2	-0.399	0.045	N/A	N/A	N/A	N/A	N/A	N/A
	GNAL	G protein subunit alpha L	N/A	N/A	N/A	N/A	0.497	0.017	N/A	N/A
	GNB2	G protein subunit beta 2	-0.631	0.038	N/A	N/A	N/A	N/A	N/A	N/A
	GNG7	G protein subunit gamma 7	N/A	N/A	N/A	N/A	0.566	0.022	N/A	N/A
	GRIA1	glutamate ionotropic receptor AMPA type subunit 1	N/A	N/A	N/A	N/A	N/A	N/A	-0.497	0.030
	GRIK1	glutamate ionotropic receptor kainate type subunit 1	N/A	N/A	N/A	N/A	N/A	N/A	-0.587	0.024
	GRIK4	glutamate ionotropic receptor kainate type subunit 4	N/A	N/A	N/A	N/A	N/A	N/A	-0.748	0.016
	GRIN2B	glutamate ionotropic receptor NMDA type subunit 2B	0.457	0.046	N/A	N/A	N/A	N/A	N/A	N/A
	GRM4	glutamate metabotropic receptor 4	-1.066	0.041	N/A	N/A	N/A	N/A	N/A	N/A
	ITPR1	inositol 1,4,5-trisphosphate receptor type 1	N/A	N/A	N/A	N/A	N/A	N/A	0.518	0.036
	MAP2K2	mitogen-activated protein kinase kinase 2	-0.704	0.040	N/A	N/A	N/A	N/A	N/A	N/A
	MRAS	muscle RAS oncogene homolog	N/A	N/A	N/A	N/A	0.359	0.049	N/A	N/A
	PIK3C2B	phosphatidylinositol-4-phosphate 3-kinase catalytic subunit type 2 beta	N/A	N/A	N/A	N/A	N/A	N/A	0.820	0.013

	PIK3R2	phosphoinositide-3-kinase regulatory subunit 2	-0.747	0.034	N/A	N/A	N/A	N/A	N/A	N/A
	PLCB4	phospholipase C beta 4	N/A	N/A	N/A	N/A	N/A	N/A	0.769	0.021
	POLR2F	RNA polymerase II subunit F	-0.613	0.046	N/A	N/A	N/A	N/A	N/A	N/A
	POLR2I	RNA polymerase II subunit I	-0.666	0.047	N/A	N/A	N/A	N/A	N/A	N/A
	POLR2L	RNA polymerase II subunit L	-0.877	0.040	N/A	N/A	N/A	N/A	N/A	N/A
	PRKCA	protein kinase C alpha	N/A	N/A	N/A	N/A	N/A	N/A	-0.696	0.015
	PRKCG	protein kinase C gamma	N/A	N/A	N/A	N/A	N/A	N/A	-0.636	0.014
	RASD1	ras related dexamethasone induced 1	N/A	N/A	N/A	N/A	-0.914	0.021	-1.400	0.006
	RASD2	RASD family member 2	N/A	N/A	N/A	N/A	0.861	0.021	1.247	0.049
**mTOR signaling**	*AKT1S1*	AKT1 substrate 1	-0.935	0.033	-0.8529	0.0487	N/A	N/A	N/A	N/A
	AKT2	AKT serinethreonine kinase 2	N/A	N/A	N/A	N/A	N/A	N/A	0.428	0.029
	DDIT4	DNA damage inducible transcript 4	-0.688	0.031	N/A	N/A	N/A	N/A	N/A	N/A
	FAU	FAU ubiquitin like and ribosomal protein S30 fusion	-0.817	0.034	N/A	N/A	N/A	N/A	N/A	N/A
	MRAS	muscle and microspikes RAS	N/A	N/A	N/A	N/A	0.359	0.049	N/A	N/A
	PIK3C2B	phosphatidylinositol-4-phosphate 3-kinase catalytic subunit type 2 beta	N/A	N/A	N/A	N/A	N/A	N/A	0.820	0.013
	PIK3R2	phosphoinositide-3-kinase regulatory subunit 2	-0.747	0.034	N/A	N/A	N/A	N/A	N/A	N/A
	PLD5	phospholipase D family member 5	N/A	N/A	N/A	N/A	N/A	N/A	0.988	0.030
	PRKCA	protein kinase C alpha	N/A	N/A	N/A	N/A	N/A	N/A	-0.696	0.015
	PRKCG	protein kinase C gamma	N/A	N/A	N/A	N/A	N/A	N/A	-0.636	0.014
	RASD1	ras related dexamethasone induced 1	N/A	N/A	N/A	N/A	-0.914	0.021	-1.400	0.006
	RASD2	RASD family member 2	N/A	N/A	N/A	N/A	0.861	0.021	1.247	0.049
	RHOB	ras homolog family member B	-0.544	0.031	N/A	N/A	N/A	N/A	N/A	N/A
	RHOBTB2	Rho related BTB domain containing 2	N/A	N/A	N/A	N/A	0.530	0.014	0.585	0.009
	RHOG	ras homolog family member G	-0.678	0.039	-0.9144	0.0437	N/A	N/A	N/A	N/A
	RPS10	ribosomal protein S10	-0.562	0.031	N/A	N/A	N/A	N/A	N/A	N/A
	RPS12	ribosomal protein S12	-0.507	0.031	N/A	N/A	N/A	N/A	N/A	N/A
	RPS13	ribosomal protein S16	-0.561	0.031	-0.5220	0.0485	N/A	N/A	N/A	N/A
	RPS16	ribosomal protein S13	-0.561	0.046	N/A	N/A	N/A	N/A	N/A	N/A
	RPS21	ribosomal protein S21	-0.763	0.031	N/A	N/A	N/A	N/A	N/A	N/A
	RPS26	ribosomal protein S26	-0.633	0.031	N/A	N/A	N/A	N/A	N/A	N/A
	RPS28	ribosomal protein S28	-0.522	0.037	N/A	N/A	N/A	N/A	N/A	N/A
	RPS29	ribosomal protein S29	-0.795	0.031	N/A	N/A	N/A	N/A	N/A	N/A
	RPS5	ribosomal protein S5	-0.577	0.048	N/A	N/A	N/A	N/A	N/A	N/A
	RPS6KA3	ribosomal protein S6 kinase A3	N/A	N/A	N/A	N/A	N/A	N/A	0.470	0.044
	RPS6KB1	ribosomal protein S6 kinase B1	0.391	0.046	N/A	N/A	N/A	N/A	N/A	N/A
	STK11	serinethreonine kinase 11	-0.527	0.047	N/A	N/A	N/A	N/A	N/A	N/A
	VEGFB	vascular endothelial growth factor B	-0.537	0.039	-0.5606	0.0485	N/A	N/A	N/A	N/A
	VEGFD	vascular endothelial growth factor D	N/A	N/A	N/A	N/A	N/A	N/A	-0.675	0.033
**Oxidative phosphorylation**	*Atp5e*	ATP synthase, H+ transporting,	-0.565	0.031	-0.6029	0.0441	N/A	N/A	N/A	N/A

		mitochondrial F1 complex epsilon subunit								
	ATP5F1D	ATP synthase F1 subunit delta	-0.790	0.031	N/A	N/A	N/A	N/A	N/A	N/A
	ATP5MC2	ATP synthase membrane subunit c locus 2	-0.664	0.035	N/A	N/A	N/A	N/A	N/A	N/A
	COX17	cytochrome c oxidase copper chaperone COX17	-0.660	0.046	N/A	N/A	N/A	N/A	N/A	N/A
	COX6A1	cytochrome c oxidase subunit 6A1	-0.637	0.035	N/A	N/A	N/A	N/A	N/A	N/A
	NDUFA11	NADH:ubiquinone oxidoreductase subunit A11	-0.598	0.043	N/A	N/A	N/A	N/A	N/A	N/A
	NDUFA13	NADH:ubiquinone oxidoreductase subunit A13	-0.666	0.032	N/A	N/A	N/A	N/A	N/A	N/A
	NDUFA2	NADH:ubiquinone oxidoreductase subunit A2	-0.754	0.033	N/A	N/A	N/A	N/A	N/A	N/A
	NDUFA6	NADH:ubiquinone oxidoreductase subunit A6	-0.442	0.046	N/A	N/A	N/A	N/A	N/A	N/A
	NDUFA7	NADH:ubiquinone oxidoreductase subunit A7	-0.656	0.034	-0.6078	0.0485	N/A	N/A	N/A	N/A
	NDUFB7	NADH:ubiquinone oxidoreductase subunit B7	-0.790	0.040	N/A	N/A	N/A	N/A	N/A	N/A
	NDUFS7	NADH:ubiquinone oxidoreductase core subunit S7	-1.008	0.031	-0.8245	0.0485	N/A	N/A	N/A	N/A
	UQCR10	ubiquinol-cytochrome c reductase, complex III subunit X	-0.598	0.041	N/A	N/A	N/A	N/A	N/A	N/A
	UQCR11	ubiquinol-cytochrome c reductase, complex III subunit XI	-0.789	0.035	N/A	N/A	N/A	N/A	N/A	N/A
	UQCRQ	ubiquinol-cytochrome c reductase complex III subunit VII	-1.033	0.031	-0.8826	0.0485	N/A	N/A	N/A	N/A
**EIF2 signaling**	*AKT2*	thymoma viral proto-oncogene 2	N/A	N/A	N/A	N/A	N/A	N/A	0.428	0.029
	FAU	FAU ubiquitin like and ribosomal protein S30 fusion	-0.817	0.034	N/A	N/A	N/A	N/A	N/A	N/A
	MAP2K2	mitogen-activated protein kinase kinase 2	-0.704	0.040	N/A	N/A	N/A	N/A	N/A	N/A
	NKX6-2	NK6 homeobox 2	-1.547	0.034	-1.2000	0.0441	N/A	N/A	N/A	N/A
	PIK3C2B	phosphatidylinositol-4-phosphate 3-kinase catalytic subunit type 2 beta	N/A	N/A	N/A	N/A	N/A	N/A	0.820	0.013
	PIK3R2	phosphoinositide-3-kinase regulatory subunit 2	-0.747	0.034	N/A	N/A	N/A	N/A	N/A	N/A
	RASD1	RAS, dexamethasone-induced 1	N/A	N/A	N/A	N/A	-0.914	0.021	-1.400	0.006
	RASD2	RASD family, member 2	N/A	N/A	N/A	N/A	0.861	0.021	1.247	0.049
	RPL13	ribosomal protein L13	-0.570	0.040	N/A	N/A	N/A	N/A	N/A	N/A
	RPL18	ribosomal protein L18	-0.536	0.041	N/A	N/A	N/A	N/A	N/A	N/A
	RPL18A	ribosomal protein L18a	-0.537	0.047	N/A	N/A	N/A	N/A	N/A	N/A
	RPL27A	ribosomal protein L27a	-0.815	0.024	-0.8089	0.0437	N/A	N/A	N/A	N/A
	RPL28	ribosomal protein L28	-0.393	0.042	N/A	N/A	N/A	N/A	N/A	N/A
	RPLP0	ribosomal protein lateral stalk subunit P0	-0.420	0.050	N/A	N/A	N/A	N/A	N/A	N/A
	RPLP2	ribosomal protein lateral stalk subunit P2	-0.926	0.035	-0.8594	0.0485	N/A	N/A	N/A	N/A
	RPS10	ribosomal protein S10	-0.562	0.031	N/A	N/A	N/A	N/A	N/A	N/A
	RPS12	ribosomal protein S12	-0.507	0.031	N/A	N/A	N/A	N/A	N/A	N/A
	RPS13	ribosomal protein S16	-0.561	0.031	-0.5220	0.0485	N/A	N/A	N/A	N/A
	RPS16	ribosomal protein S13	-0.561	0.046	N/A	N/A	N/A	N/A	N/A	N/A
	RPS21	ribosomal protein S21	-0.763	0.031	N/A	N/A	N/A	N/A	N/A	N/A

	RPS26	ribosomal protein S26	-0.633	0.031	N/A	N/A	N/A	N/A	N/A	N/A
	RPS28	ribosomal protein S28	-0.522	0.037	N/A	N/A	N/A	N/A	N/A	N/A
	RPS29	ribosomal protein S29	-0.795	0.031	N/A	N/A	N/A	N/A	N/A	N/A
	RPS5	ribosomal protein S5	-0.577	0.048	N/A	N/A	N/A	N/A	N/A	N/A
	SREBF1	sterol regulatory element binding transcription factor 1	-0.987	0.020	-0.8347	0.0437	N/A	N/A	N/A	N/A
**cAMP-mediated signaling**	*ADCY5*	adenylate cyclase 5	N/A	N/A	N/A	N/A	0.546	0.028	N/A	N/A
	ADORA2A	adenosine A2a receptor	N/A	N/A	N/A	N/A	0.759	0.019	N/A	N/A
	ADRA2C	adrenergic receptor, alpha 2c	-0.658	0.040	N/A	N/A	N/A	N/A	N/A	N/A
	AKAP12	A-kinase anchoring protein 12	N/A	N/A	N/A	N/A	N/A	N/A	0.424	0.041
	AKAP13	A-kinase anchoring protein 13	N/A	N/A	N/A	N/A	-0.680	0.010	-0.828	0.010
	AKAP5	A-kinase anchoring protein 5	N/A	N/A	N/A	N/A	0.446	0.023	N/A	N/A
	CAMK2G	calciumcalmodulin dependent protein kinase II gamma	N/A	N/A	N/A	N/A	N/A	N/A	0.421	0.043
	CHRM4	cholinergic receptor muscarinic 4	N/A	N/A	N/A	N/A	N/A	N/A	0.852	0.045
	CHRM5	cholinergic receptor muscarinic 5	N/A	N/A	N/A	N/A	-1.185	0.022	-1.408	0.027
	DRD1	dopamine receptor D1	N/A	N/A	N/A	N/A	0.724	0.020	N/A	N/A
	DRD5	dopamine receptor D5	N/A	N/A	N/A	N/A	N/A	N/A	-1.117	0.033
	DUSP1	dual specificity phosphatase 1	N/A	N/A	N/A	N/A	N/A	N/A	0.926	0.022
	GNAI2	guanine nucleotide binding protein (G protein), alpha inhibiting 2	-0.399	0.045	N/A	N/A	N/A	N/A	N/A	N/A
	GNAL	G protein subunit alpha L	N/A	N/A	N/A	N/A	0.497	0.017	N/A	N/A
	GRM4	glutamate receptor, metabotropic 4	-1.066	0.041	N/A	N/A	N/A	N/A	N/A	N/A
	HTR1A	5-hydroxytryptamine receptor 1A	N/A	N/A	N/A	N/A	-0.584	0.043	-1.048	0.002
	Htr5b	5-hydroxytryptamine (serotonin) receptor 5B	N/A	N/A	N/A	N/A	N/A	N/A	-1.445	0.016
	MAP2K2	mitogen-activated protein kinase kinase 2	-0.704	0.040	N/A	N/A	N/A	N/A	N/A	N/A
	PDE10A	phosphodiesterase 10A	N/A	N/A	N/A	N/A	0.916	0.022	N/A	N/A
	PDE11A	phosphodiesterase 11A	N/A	N/A	N/A	N/A	N/A	N/A	-1.205	0.040
	PDE1B	phosphodiesterase 1B	N/A	N/A	N/A	N/A	0.586	0.026	N/A	N/A
	PDE7B	phosphodiesterase 7B	N/A	N/A	N/A	N/A	0.703	0.010	1.038	0.025
	PDE8B	phosphodiesterase 8B	N/A	N/A	N/A	N/A	N/A	N/A	0.530	0.018
	PKIG	protein kinase inhibitor, gamma	-0.591	0.042	N/A	N/A	N/A	N/A	N/A	N/A
	RGS4	regulator of G protein signaling 4	N/A	N/A	N/A	N/A	N/A	N/A	0.853	0.014
**DARPP-32 signaling**	*ADCY5*	adenylate cyclase 5	N/A	N/A	N/A	N/A	0.546	0.028	N/A	N/A
	CALY	calcyon neuron-specific vesicular protein	-0.599	0.034	N/A	N/A	N/A	N/A	N/A	N/A
	CAMKK2	calciumcalmodulin dependent protein kinase kinase 2	N/A	N/A	N/A	N/A	0.386	0.048	0.567	0.009
	DRD1	dopamine receptor D1	N/A	N/A	N/A	N/A	0.724	0.020	N/A	N/A
	DRD5	dopamine receptor D5	N/A	N/A	N/A	N/A	N/A	N/A	-1.117	0.033
	GNAI2	guanine nucleotide binding protein (G protein), alpha inhibiting 2	-0.399	0.045	N/A	N/A	N/A	N/A	N/A	N/A
	GRIN2B	glutamate receptor, ionotropic, NMDA2B (epsilon 2)	0.457	0.046	N/A	N/A	N/A	N/A	N/A	N/A
	GRIN3A	glutamate ionotropic receptor NMDA type subunit 3A	N/A	N/A	N/A	N/A	N/A	N/A	-0.441	0.023

	ITPR1	inositol 1,4,5-trisphosphate receptor type 1	N/A	N/A	N/A	N/A	N/A	N/A	0.518	0.036
	KCNJ11	potassium inwardly rectifying channel subfamily J member 11	N/A	N/A	N/A	N/A	N/A	N/A	0.534	0.039
	KCNJ4	potassium inwardly rectifying channel subfamily J member 4	-0.869	0.036	N/A	N/A	0.550	0.022	N/A	N/A
	PLCB4	phospholipase C beta 4	N/A	N/A	N/A	N/A	N/A	N/A	0.769	0.021
	PPP1R14A	protein phosphatase 1, regulatory (inhibitor) subunit 14A	-0.940	0.041	N/A	N/A	N/A	N/A	N/A	N/A
	PPP1R14B	protein phosphatase 1, regulatory (inhibitor) subunit 14B	-0.705	0.044	N/A	N/A	N/A	N/A	N/A	N/A
	PPP1R1B	protein phosphatase 1 regulatory inhibitor subunit 1B	N/A	N/A	N/A	N/A	0.823	0.022	N/A	N/A
	PRKCA	protein kinase C alpha	N/A	N/A	N/A	N/A	N/A	N/A	-0.696	0.015
	PRKCG	protein kinase C gamma	N/A	N/A	N/A	N/A	N/A	N/A	-0.636	0.014
**GABA receptor signaling**	*ADCY5*	adenylate cyclase 5	N/A	N/A	N/A	N/A	0.546	0.028	N/A	N/A
	AP2S1	adaptor related protein complex 2 subunit sigma 1	-0.595	0.046	N/A	N/A	N/A	N/A	N/A	N/A
	CACNA1H	calcium voltage-gated channel subunit alpha1 H	-0.687	0.046	N/A	N/A	N/A	N/A	N/A	N/A
	CACNA1I	calcium voltage-gated channel subunit alpha1 I	N/A	N/A	N/A	N/A	N/A	N/A	0.554	0.020
	CACNG3	calcium voltage-gated channel auxiliary subunit gamma 3	N/A	N/A	N/A	N/A	0.449	0.028	N/A	N/A
	CACNG4	calcium voltage-gated channel auxiliary subunit gamma 4	N/A	N/A	N/A	N/A	0.519	0.023	N/A	N/A
	CACNG7	calcium voltage-gated channel auxiliary subunit gamma 7	-0.523	0.047	N/A	N/A	N/A	N/A	N/A	N/A
	CACNG8	calcium voltage-gated channel auxiliary subunit gamma 8	-0.648	0.043	N/A	N/A	N/A	N/A	N/A	N/A
	GABRA2	gamma-aminobutyric acid type A receptor alpha2 subunit	N/A	N/A	N/A	N/A	N/A	N/A	-0.569	0.030
	GABRA5	gamma-aminobutyric acid type A receptor alpha5 subunit	N/A	N/A	N/A	N/A	-0.615	0.022	-1.078	0.003
	GABRB1	gamma-aminobutyric acid type A receptor beta1 subunit	0.555	0.049	N/A	N/A	N/A	N/A	-0.453	0.021
	GABRD	gamma-aminobutyric acid type A receptor delta subunit	N/A	N/A	N/A	N/A	0.674	0.017	0.993	0.010
	GPR37	G protein-coupled receptor 37	-0.976	0.026	-0.8489	0.0485	N/A	N/A	N/A	N/A
	MRAS	muscle RAS oncogene homolog	N/A	N/A	N/A	N/A	0.359	0.049	N/A	N/A
	SLC32A1	solute carrier family 32 member 1	-1.206	0.024	-1.0810	0.0485	N/A	N/A	N/A	N/A

List of genes annotated with the top ten canonical signaling pathways affected in susceptible and resilient subgroups of MIA-exposed offspring. Ingenuity Pathway Analysis (IPA) was used to identify canonical signaling pathways affected in the medial prefrontal cortex (mPFC) and amygdala (Amy) of susceptible (Sus-POL) and resilient (Res-POL) subgroups of MIA-exposed offspring relative to reference control (Ref-CON) offspring. For either brain region, the list denotes the top ten signaling pathways and the differentially expressed genes (DEGs) annotated with each pathway. The direction of transcriptional changes is shown in terms of log2 fold changes (Log2FC). Only genes that passed a false discovery rate (FDR) correction set at a 5% threshold (*q* < 0.05) were considered as DEGs and included in the list. Genes that did not pass this FDR threshold are denoted as N/A (not applicable). A graphical summary of the top ten canonical

signaling pathways affected in Sus-POL and Res-POL offspring is provided in *Figure 3* of the main text. The full list of genes has been deposited at the NCBI's Gene Expression Omnibus and is accessible through GEO series accession number GSE15048.

Supplemental Table S3

Sus-POL over Res-POL (mPFC)			
**Gene Symbol**	**Gene Name**	**Log2FC**	***q*-value**
ARC	activity regulated cytoskeleton associated protein	-0.838	0.00539
FOS	fos proto-oncogene, AP-1 transcription factor subunit	-0.891	0.00517
NPAS4	neuronal PAS domain protein 4	-0.771	0.02633
NR4A1	nuclear receptor subfamily 4 group A member 1	-0.704	0.00517

List of genes annotated with the term "neuronal activity–regulated gene transcription" in susceptible relative to resilient MIA-exposed offspring. Ingenuity Pathway Analysis (IPA) was used to identify signaling pathways affected in the medial prefrontal cortex (mPFC) of susceptible (Sus-POL) relative to resilient (Res-POL) MIA-exposed offspring. The direction of transcriptional changes is shown in terms of log2 fold changes (Log2FC). Only genes that passed a false discovery rate (FDR) correction set at a 5% threshold ($q < 0.05$) were considered as DEGs and included in the list.

Supplemental Table S4

Sus-POL over Res-POL (Amy)			
**Gene Symbol**	**Gene Name**	**Log2FC**	**q-value**
ASPA	aspartoacylase	-0.853	0.04957
CLDN11	claudin 11	-0.514	0.04234
DAAM2	dishevelled associated activator of morphogenesis 2	-0.431	0.04234
ERBB3	erb-b2 receptor tyrosine kinase 3	-0.576	0.04443
FA2H	fatty acid 2-hydroxylase	-0.590	0.04009
HPCAL1	hippocalcin like 1	0.752	0.04957
LPAR1	lysophosphatidic acid receptor 1	-0.484	0.04234
MAG	myelin associated glycoprotein	-0.665	0.04957
MBP	myelin basic protein	-0.580	0.04957
NDRG1	N-myc downstream regulated 1	-0.430	0.04234
NOS1	nitric oxide synthase 1	0.466	0.04135
PLP1	proteolipid protein 1	-0.666	0.04009
PMP22	peripheral myelin protein 22	-0.663	0.04234
PVALB	parvalbumin	-1.323	0.02493
SYN2	synapsin II	0.446	0.04367
SYT2	synaptotagmin 2	-1.152	0.04957
TF	transferrin	-0.494	0.04135
UGT8	UDP glycosyltransferase 8	-0.604	0.02493
VAMP1	vesicle associated membrane protein 1	-0.634	0.04135

List of genes annotated with the terms "schizophrenia", "demyelination", "abnormal morphology of myelin sheath", "hypomyelination of axons" and "abnormal morphology of

oligodendrocytes" in susceptible relative to resilient MIA-exposed offspring. Ingenuity Pathway Analysis (IPA) was used to identify signaling pathways affected in the amygdala (Amy) of susceptible (Sus-POL) relative to resilient (Res-POL) MIA-exposed offspring. The direction of transcriptional changes is shown in terms of log2 fold changes (Log2FC). Only genes that passed a false discovery rate (FDR) correction set at a 5% threshold ($q < 0.05$) were considered as DEGs and included in the list. A graphical representation of the genes annotated with the term "schizophrenia" is provided in ***Suppl. Fig. S4a***, whereas genes annotated with the terms "demyelination", "abnormal morphology of myelin sheath", "hypomyelination of axons" and "abnormal morphology of oligodendrocytes" are graphically summarized in ***Suppl. Fig. S4b***.

Supplemental Figure S4

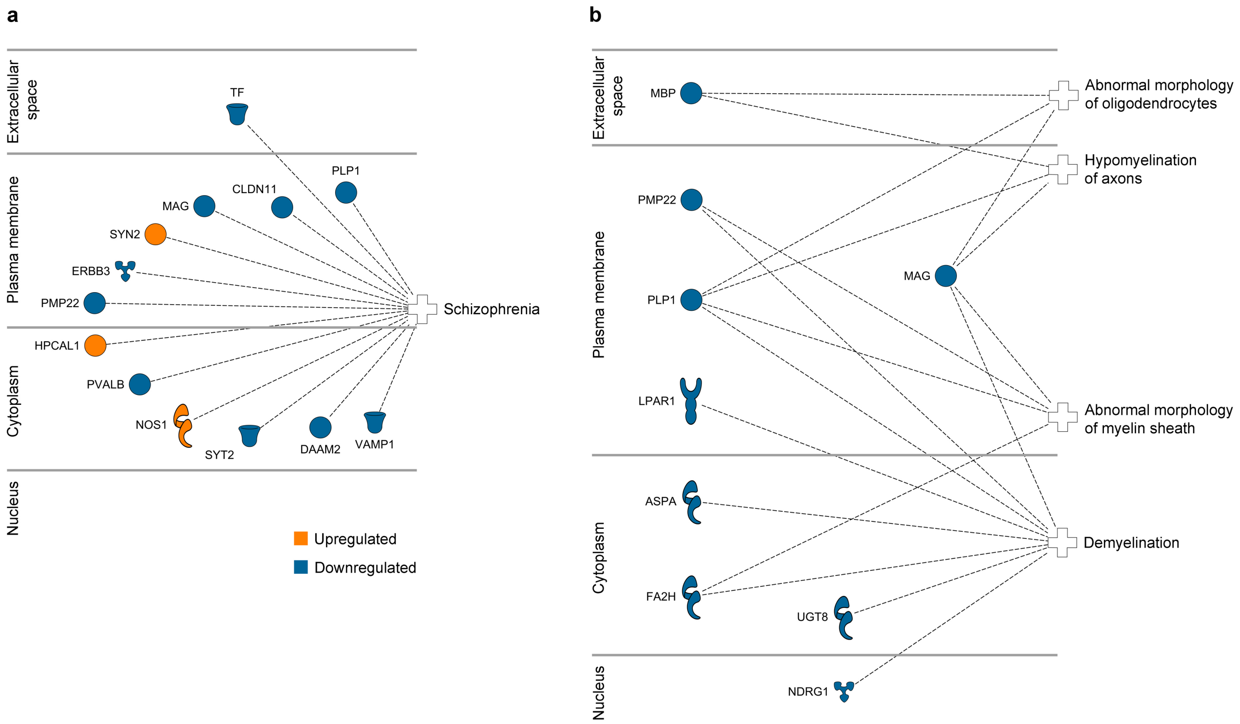

Schematic representation of transcriptional changes occurring in the amygdala of susceptible relative to resilient MIA-exposed offspring. Ingenuity Pathway Analysis (IPA) was used to identify pathways affected in susceptible (Sus-POL) relative to resilient (Res-POL) MIA-exposed offspring. Upregulated and downregulated genes are shown in orange and blue, respectively. The magnitude of the transcriptional changes are summarized in terms of log2 fold changes (Log2FC) and *q*-values in ***Suppl. Table S4***. For each gene, the figure also specifies the subcellular location of the corresponding protein. **(a)** Graphical representation of the genes annotated with the term "schizophrenia". **(b)** Graphical representation of the genes annotated with the terms "demyelination", "abnormal morphology of myelin sheath", "hypomyelination of axons" and "abnormal morphology of oligodendrocytes".

Supplemental Figure S5

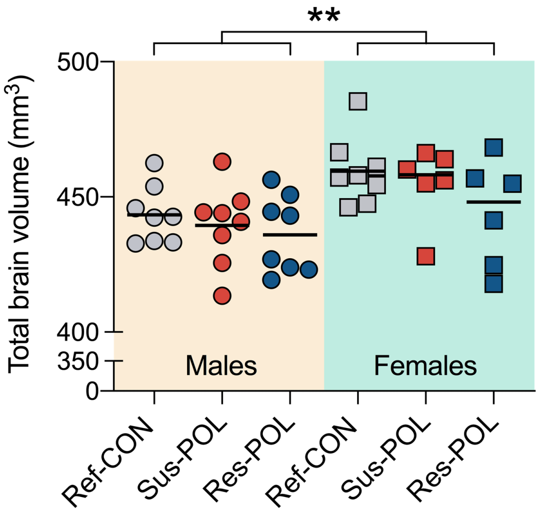

Total brain volumes of male and female offspring of the resilient and susceptible subgroups of MIA-exposed and control groups. Total brain volume, as measured by ex-vivo MRI, did not vary as a function of susceptibility or resilience to prenatal poly(I:C) exposure, but generally differed as a function of sex. **$p < 0.01$, reflecting the main effect of sex in the 3 × 2 (group × sex) ANOVA of total brain volume. Ref-CON, reference control offspring (*n* = 16; 8 m, 8 f); Res-POL, resilient subgroup of poly(I:C)-exposed offspring (*n* = 14; 8 m, 6 f); Sus-POL, susceptible subgroup of poly(I:C)-exposed offspring (*n* = 14; 8 m, 6 f).

Supplemental Table S5

	Ref-CON		Res-POL		Sus-POL				Ref-CON vs. Res-POL		Ref-CON vs. Sus-POL		Res-POL vs. Sus-POL	
**Atlas ROI**	**Mean**	**SD**	**Mean**	**SD**	**Mean**	**SD**	***p*-value**	***q*-value**	**% change**	**Effect**	**% change**	**Effect**	**% change**	**Effect**
Retrosplenial cortex	9.60	0.31	9.25	0.60	9.19	0.27	0.02	0.59	-3.58	-1.10	-4.20	-1.29	-0.65	-0.10
Red nucleus	0.68	0.03	0.64	0.03	0.67	0.03	0.01	0.59	-5.27	-1.08	-1.69	-0.35	3.78	0.71
Hypothalamus	11.55	0.45	11.07	0.47	11.44	0.59	0.04	0.59	-4.13	-1.07	-0.96	-0.25	3.31	0.78
Inferior colliculus	4.31	0.19	4.11	0.24	4.20	0.18	0.04	0.59	-4.45	-1.00	-2.36	-0.53	2.18	0.38

Brain volumetric changes in exploratory analyses of ex-vivo MRI. The table summarizes the four atlas region of interests (ROIs), which differed at the group level at an exploratory threshold ($p < 0.05$ uncorrected). For each ROI and group (Res-CON, Res-POL and Sus-POL), the mean + SD of absolute volume (mm^3) are provided, along with the relative changes (in %) between groups and their effect sizes (in units of SD). The *p*-values are derived from one-way ANOVA models and the *q*-values represent the FDR-corrected *p*-value. Ref-CON, reference control offspring (*n* = 16; 8 m, 8 f); Res-POL, resilient subgroup of poly(I:C)-exposed offspring (*n* = 14; 8 m, 6 f); Sus-POL, susceptible subgroup of poly(I:C)-exposed offspring (*n* = 14; 8 m, 6 f).

Supplemental Figure S7

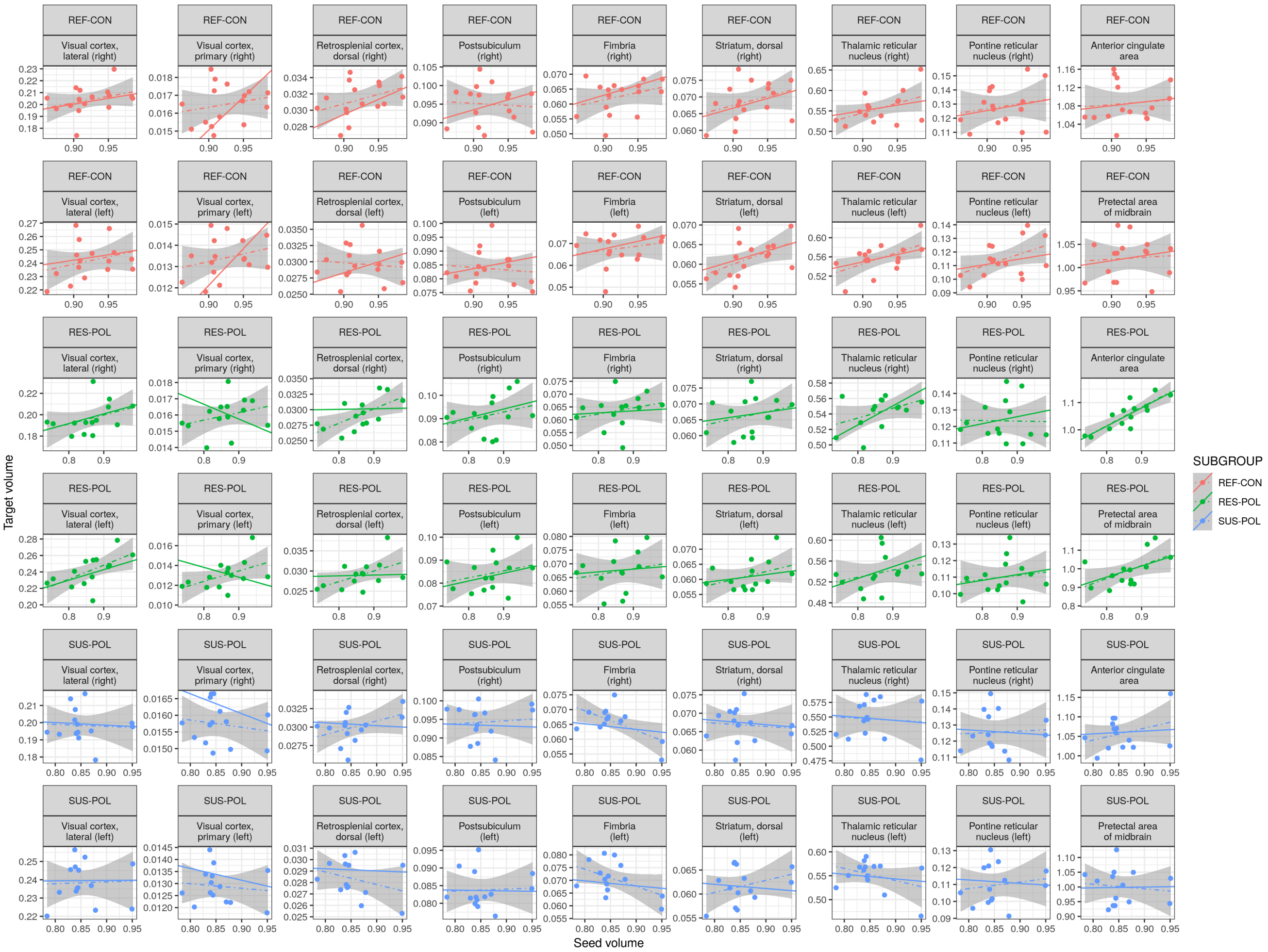

Summary of structural covariance data. The graph shows the structural covariance between seed and target, for all 18 target regions, separated by groups. REF-CON, reference control offspring (n = 16; 8 m, 8 f); RES-POL, resilient subgroup of poly(**I**:C)-exposed offspring (n = 14; 8 m, 6 f); SUS-POL, susceptible subgroup of poly(**I**:C)-exposed offspring (n = 14; 8 m, 6 f).
